# Applying blood-derived epigenetic algorithms to saliva: cross-tissue similarity of DNA-methylation indices of aging, physiology, and cognition

**DOI:** 10.1186/s13148-025-01868-2

**Published:** 2025-04-23

**Authors:** Sepideh Zarandooz, Laurel Raffington

**Affiliations:** Max Planck Research Group Biosocial – Biology, Social Disparities, and Development, Max Planck Institute for Human Development, Berlin, Germany

**Keywords:** DNA methylation, Biological aging, Cross-tissue similarity, Saliva, Blood

## Abstract

**Background:**

Epigenetic algorithms of aging, health, and cognition, based on DNA-methylation (DNAm) patterns, are prominent tools for measuring biological age and have been linked to age-related diseases, cognitive decline, and mortality. While most of these methylation profile scores (MPSs) are developed in blood tissue, there is growing interest in using less invasive tissues like saliva. The aim of the current study is to probe the cross-tissue intraclass correlation coefficients (ICCs) of MPSs developed in blood applied to saliva DNAm from the same people. While our primary focus is on MPSs that were previously found to be robustly correlated with social determinants of health, including second- and third-generation clocks and MPSs of physiology and cognition, we also report ICC values for first-generation clocks to enable comparison across metrics. We pooled three publicly available datasets that had both saliva and blood DNAm from the same individuals (total *n* = 107, aged 5–74 years), corrected MPSs for cell composition within each tissue, and computed the cross-tissue ICCs.

**Results:**

We found that after correcting for cell composition, saliva–blood cross-tissue ICCs were moderate for second- and third-generation indices of aging and MPSs of physiology and cognition. Specifically, PCGrimAge had the highest ICC (0.76), followed by PCPhenoAge (0.72), a measure of cognitive performance (Epigenetic-*g*, 0.69), DunedinPACE (0.68), PCGrimAge Acceleration (0.67), PCPhenoAge Acceleration (0.66), an MPS of hs-CRP (0.58), and BMI (0.54). These ICCs appear lower than previous reports on within-tissue ICCs (saliva ICCs range from 0.67 to 0.85, blood ICCs range from 0.73 to 0.93). Cross-tissue ICCs values for first-generation biological age acceleration measures were poor, ranging from 0.19 to 0.25.

**Conclusions:**

Our findings suggest that applying second- and third-generation MPSs of biological age acceleration and related phenotypes developed in blood to saliva DNAm results in moderate cross-tissue similarity and the precise cross-tissue correspondence differs by measure. While the degree of cross-tissue similarity of several MPSs may suffice for some research settings, it may not be suitable in clinical or commercial applications. Collection of both blood and saliva DNAm samples is necessary to validate existing algorithms and to customize MPSs in saliva DNAm.

**Supplementary Information:**

The online version contains supplementary material available at 10.1186/s13148-025-01868-2.

## Introduction

Epigenetic algorithms of aging, health, and cognition, based on DNA-methylation (DNAm) patterns, are prominent tools for measuring biological age and have been linked to age-related diseases, cognitive decline, and mortality [1–4]. While most of these methylation profile scores (MPSs) are developed using blood tissues—which is considered the gold standard—there is growing interest in using less invasive tissues like saliva, cheek, and dried-blood spot DNA, as it is less invasive, can be sampled via postal kits, does not require trained medical professionals, and can result in higher participation rates than blood (saliva 72% vs. blood 31%) [5]. DNAm patterns, which encode cell identity, vary by tissue types. Blood samples consist of 100% immune cells, saliva samples consist of approximately ~ 65% immune cells and ~ 35% epithelial cells [6]. Because distinct cell types in blood and saliva exhibit different methylation patterns, estimating these cell-type proportions and adjusting for their variation is crucial for epigenetic clock and epigenome-wide association studies [7–9]. This approach helps to distinguish between effects caused by differences in cell-type composition and DNAm differences occurring in specific cell types.

Probing the similarity of MPSs between saliva and blood DNAm is particularly relevant for pediatric studies, because they are substantially more likely to collect DNA from saliva than blood. A recent meta-analysis on MPSs of biological aging finds that the proportion of studies using saliva compared to blood is 50% and 37.5% in pediatric samples, respectively, and 3.4% and 94.1% in adult studies (Rezaki, Willems et al., in progress). In line with the notion that adult health and psychological functioning has roots in childhood, these studies have found that children living in under resourced social contexts tend to have saliva MPSs associated with accelerated biological age, a faster pace of aging, higher chronic inflammation, higher body mass index, and lower cognitive performance in adults [10, 11] (for review see [12]). Moreover, racially marginalized children tend to have accelerated longitudinal increases in biological aging from 9 to 15 years [13]. Nascent studies have also explored the clinical relevance of MPSs in pediatric cohorts, such as helping to identify children at increased risk for early-onset obesity and morbidity [14]. However, uncertainty about the potential loss of signal in saliva MPSs remains an important methodological concern [12].

Here, we pooled three publicly available datasets that had both saliva and blood DNAm from the same individuals (total *n* = 107, age range 5–74 years), corrected MPSs for cell composition within each tissue, and computed the meta-analyzed cross-tissue intraclass correlation coefficients (ICCs). Our focus is on MPSs developed in adults that were previously reported to be sensitive to social determinants of health in both adults and children and correlated with developmental phenotypes ascertained in child DNAm samples [13, 15–17]. This includes MPSs of accelerated biological age (PC-based and non-PC-based PhenoAge [18] and GrimAge Acceleration [19]), the pace of biological aging (DunedinPACE [20]), an indicator of chronic systemic inflammation (DNAm-CRP) [21], body mass index (DNAm-BMI [22]), and general cognitive performance (Epigenetic-*g*) [23]. To enable comparison across studies, we also report results for first-generation epigenetic clocks developed from analyses of age differences in DNAm (Horvath, Horvath Skin and Blood Acceleration, and Hannum [24–26]). We further examine whether the choice of cell composition estimation method affects cross-tissue similarity and focus on two of the most widely used statistical methods (reference-free versus reference-based [27, 28]). We compare our cross-tissue results to previous studies reporting ICCs of MPSs from duplicate samples collected from the same people within the same tissue [2, 16, 17, 29, 30], because such duplicate samples were not available in our datasets.

## Methods

### Participants

We pooled three publicly available datasets in the Gene Expression Omnibus (GEO) database under their respective accession numbers. Dataset1 (GSE111165) includes 21 subjects (7 female, 33%) with medically intractable epilepsy from the University of Iowa Hospitals and Clinics, USA, aged from 5 to 61 years (mean 30.95 years, SD = 16.39) [31]. In this dataset, blood samples were typically taken at the end of surgery in the operating room, and saliva samples were collected two days after the operation. Dataset2 (GSE130153) comprises samples from 22 healthy participants from Japan (7 female, 32%) aged 23 to 40 years (mean = 30.68 years, SD = 5.45) [32]. Dataset3 (GSE61653) includes samples from 64 healthy African-American adults (53 female, 82%) aged 20 to 74 years (mean = 41.35 years, SD = 11.53) [33, 34]. For the second and third datasets, saliva and blood samples appear to have been collected simultaneously.

### DNA and DNA-methylation extraction

The three studies differed in their DNA extraction and DNA-methylation profiling. For DNA extraction, GSE111165 used the MasterPure™ DNA Extraction Kit, while GSE130153 applied the Oragene DNA Collection Kit and the prepIT-C2D Genomic DNA MiniPrep Kit. GSE61653 used the Puregene Genomic DNA Kit. For DNA-methylation extraction, GSE130153 utilized the Infinium HumanMethylation450k and GSE61653 and GSE111165 the EPIC BeadChip™ Kit.

DNA-methylation data were preprocessed using a variety of software tools that varied between datasets, including Minfi [35] and RnBeads [36], CpGassoc [37], and Illumina’s BeadStudio. Quality control measures were applied to all datasets, including removal of samples and probes based on criteria such as call rates, average intensity values, percentage of missing data, and the presence of known SNPs or cross-hybridization DNAm sites. Studies GSE111165 and GSE61653 used the beta mixture quantile dilation (BMIQ) method [38] for sample normalization. Further details on these methods can be found in the respective publications [31, 32, 34].

### Methylation profile scores

We computed MPSs in each cohort by applying published DNAm algorithms described in Table 1.

**Table 1:** Description of methylation profile scores

Measure	Description
*First-generation clocks*	
Horvath acceleration	The Horvath clock was developed as a multi-tissue predictor of biological age using 8,000 samples from 82 DNA-methylation datasets across 51 healthy tissues and cell types. The clock is based on 353 CpG sites and is calculated using a penalized regression model to define DNA-methylation age [24]. Horvath was residualized for chronological age to compute Horvath Acceleration
Horvath skin and blood acceleration	The Horvath Skin and Blood clock is a DNA-methylation age estimator developed from 391 CpG sites, designed to measure biological age in human fibroblasts, keratinocytes, endothelial cells, and various tissue types including skin and blood. This clock shows strong age correlations across multiple cells types and predicts lifespan and correlates with age-related health conditions [25]. Horvath Skin and Blood clock was residualized for chronological age to compute Horvath Skin and Blood Acceleration
Hannum acceleration	The Hannum clock is designed to measure human aging rates using over 450,000 CpG markers from whole blood samples of 656 individuals aged 19 to 101. This model captures the rate at which an individual’s methylome ages, taking into account factors such as gender and genetic variants. It reveals differences in aging rates that contribute to epigenetic drift and are reflected in the transcriptome, with applications across multiple tissues [26]. Hannum was residualized for chronological age to compute Hannum Acceleration
*Second-generation clocks*	
GrimAge acceleration	GrimAge, an estimator of mortality risk, was originally developed using age, sex, and DNA-methylation smoking history in the Framingham Heart Study Offspring cohort aged 53–73 years [19]. To increase reliability, PCGrimAge was developed using DNA-methylation principal components (PCs) derived to capture major variations in the DNA-methylation data, and then calculated from these PCs using elastic-net regression as described in Higgins-Chen et al.(2022) [50, 56]. PCGrimAge was residualized for chronological age and sex to compute PCGrimAge Acceleration. The PC code and necessary resources are available on the GitHub repository: https://github.com/MorganLevineLab/PC-Clocks/
PhenoAge acceleration	PhenoAge, derived from physiological indicators and chronological age in the InCHIANTI Study (21–100 years) [18], was subsequently modeled using DNA methylation. To improve reliability, PhenoAge was calculated using DNA-methylation principal components (PCs) which were derived to capture major variations in the DNA-methylation data. PCPhenoAge was then calculated from these PCs using elastic-net regression, as detailed in Higgins-Chen et al. (2022) [50, 56]. PhenoAge Acceleration was calculated by residualizing PC-based and CpG-based PhenoAge for chronological age. The PC code and necessary resources are available on the GitHub repository: https://github.com/MorganLevineLab/PC-Clocks/
*Third-generation clocks*	
DunedinPACE	DunedinPACE-pace of aging was developed to quantify change over 19 years of follow-up in 19 system-integrity biomarkers, including hs-CRP and BMI (repeated at ages 26, 32, 38, and 45 years) in the Dunedin Study birth cohort [20]. DunedinPACE increments represent years of physiological change per chronological year. A value of 1 reflects the average rate of aging in the cohort between the ages of 26 and 45. A value of 1.01 indicates an aging rate that is 1% faster than the average [50]. DunedinPACE was calculated using a published algorithm, available at https://github.com/danbelsky/DunedinPACE/ [57]
*Cross-sectional physiology*	
DNAm-CRP	DNAm-CRP was computed using the summary results of an epigenome-wide association study of serum hs-C-reactive protein (CRP), a sensitive marker of low-grade inflammation, in adults from multiple cohorts with mean ages 60–87 years controlling for age, sex, and BMI [21]. DNAm-CRP scores were derived from the sum of the products of the weight and the corresponding beta estimate for each participant across the 218 CpG sites found to be significantly associated with CRP in the EWAS. Higher score indicates a DNAm profile that more closely resembles the DNAm profile of adults with higher hs-CRP
DNAm-BMI	DNAm-BMI was computed using the summary results of an epigenome-wide association study of body mass index in adults from multiple cohorts at high risk of obesity aged 51 to 70 controlling for age and sex [22]. DNAm-BMI scores were derived from the sum of the products of the weight and the corresponding beta estimate for each participant across the 278 CpG sites found to be significantly associated with BMI in the EWAS. A higher score is indicative of a DNAm profile that is more similar to the DNAm profile of adults from multiple cohorts and ancestries with a higher BMI
*Cognitive performance*	
Epigenetic-*g*	Epigenetic-*g* was developed to quantify general cognitive functioning in adults from the Generation Scotland Study ages 18 to 93 controlling for age, sex, and BMI. The algorithm sums the products of the weight and the corresponding beta estimate for each participant across more than 750 000 CpG sites [23]. It is available at https://gitlab.com/danielmccartney/ewas_of_cognitive_function. In line with this algorithm, methylation values at each CpG site were normalized to have a mean of 0 and a SD of 1 before computing the profile score [17]. A higher score indicates a DNAm profile that is more closely similar to the DNAm profile of adults in the Generation Scotland Study who scored higher on tests of general cognitive function, including logical memory, digit symbol test score, verbal fluency, and vocabulary

### Cell composition estimation

We estimated blood and saliva cell composition directly based on methylation data in two ways: First, we estimated five cell types using the “RefFreeEWAS” [39] *R* package (*i.e.,* reference-free cell composition [28]). Second, we used sample-specific reference values to estimate the proportion of leukocyte and epithelial cells in both blood and saliva (*i.e.,* reference-based cell composition). The “Reinius” [40] dataset was used as the reference for blood samples and the “Saliva” [6] dataset was used as the reference for saliva samples. These estimations were performed using the *R* package “ewastools” [41], which is based on the Houseman algorithm [27]. We used both methods to improve reliability, as reference-based methods can be limited by the representativeness of the reference, whereas reference-free methods offer flexibility in different contexts.

### Statistical analysis

Our statistical analyses proceeded in six steps. First, MPSs listed in Table 1 were computed separately in saliva and blood samples within each cohort. Second, MPSs were residualized for technical factors (array, batch) and cell composition separately in saliva and blood samples within each cohort. We distinguish between reference-free and reference-based cell composition correction, which are among the most common approaches for estimating cell-type composition from DNAm data [42] (see “Cell composition estimation”). Third, we computed cross-tissue similarity within each cohort by calculating ICCs between saliva and blood MPSs for each measure. Fourth, we performed a random-effects meta-analysis using the DerSimonian and Laird (DL) method to test whether ICC values differed significantly between datasets [43]. The ICCs from the three datasets served as the random effects in this analysis, which was performed using the "metafor" package in *R* and applied to all MPSs. Fifth*,* we assessed the effect of cell composition estimation methods on cross-tissue ICCs in a mixed-effects meta-regression, where the ICC is regressed on the cell composition estimation method (reference-based versus reference-free). The cell estimation method is treated as a fixed effect and cohort as a random effect variable. We report the meta-analyzed results using the reference-free cell composition correction unless noted otherwise. Sixth, we probed whether ICCs differed by chronological age in a linear regression where saliva MPS was regressed on blood MPS, age, and an interaction term of blood MPSs and age. We consider ICC values below 0.50 as poor, between 0.50 and 0.75 as moderate, between 0.75 and 0.90 as good, and above 0.90 as excellent, following the guideline that offers a comprehensive framework for selecting and reporting ICCs in reliability research [44].

## Results

**Fig. 1:**
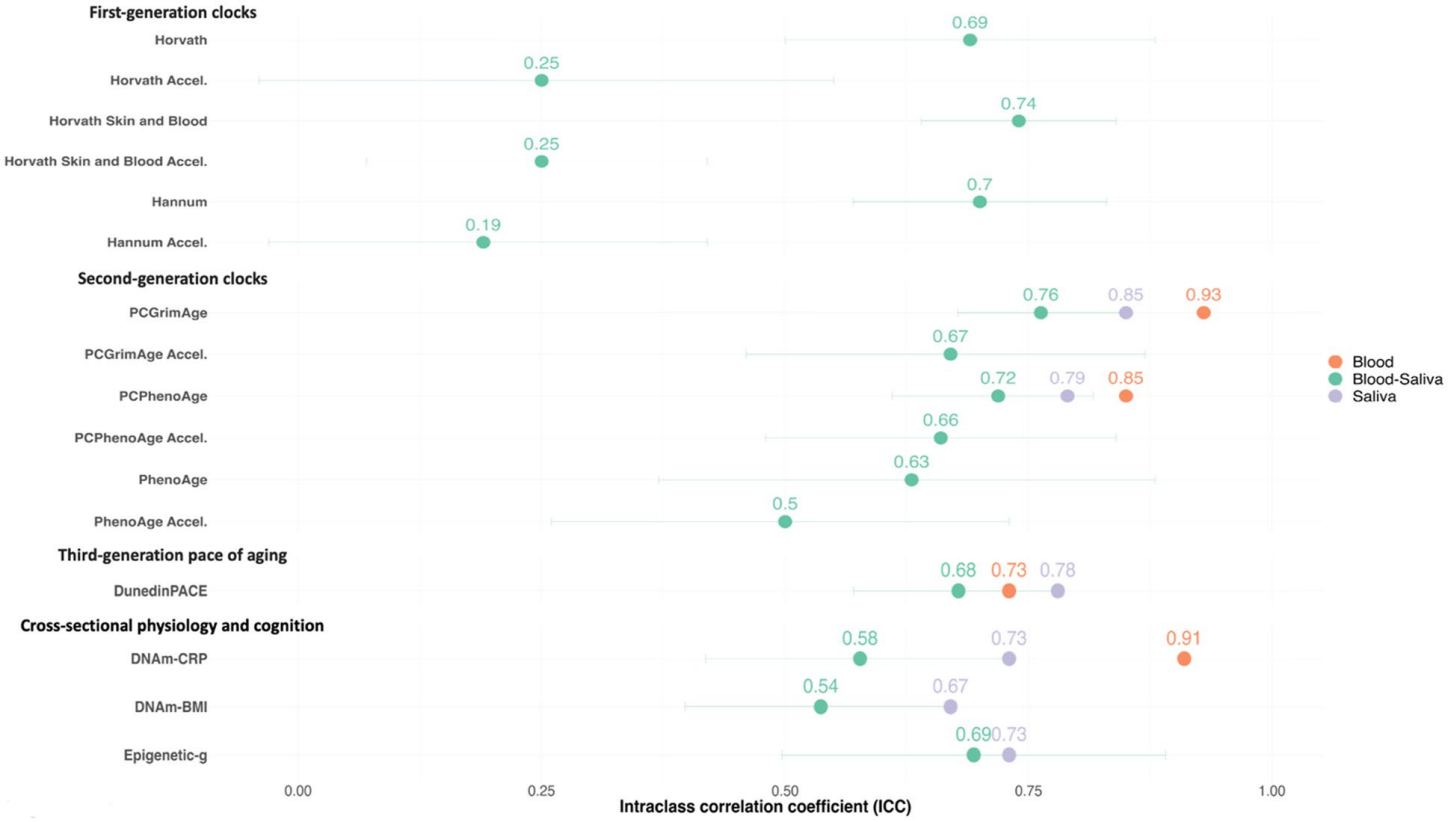
Cross-tissue and within-tissue similarity of DNA-methylation measures of aging, cross-sectional physiology, and cognition. This figure depicts intraclass correlation coefficients (ICCs) and 95% confidence intervals of DNA-methylation profile scores developed in blood. The green bars depict the cross-tissue ICCs meta-analyzed across three cohorts (*n* = 107) in the present study. The orange point estimates depict the ICCs reported in previous studies with duplicate samples of blood collected in the same people, where available. The purple point estimates depict the ICCs reported in previous studies with duplicate samples of saliva collected in the same people, where available. The blood ICC values for PCGrimAge, PCPhenoAge, and DunedinPACE are from Reed et al. (2022) [2], and the ICC value for DNAm-CRP (a marker of chronic inflammation) is from Schmunk et al., 2023 [29]. The saliva ICCs are all from child and adolescent samples, specifically ICCs for PCGrimAge, PCPhenoAge, DunedinPACE, and Epigenetic-*g* are from deSteiguer et al. (2023) [30], the ICC value for DNAm-CRP is from Raffington et al. (2022) [17], and DNAm-BMI (body mass index) is from Raffington et al. (2023) [16]. “Accel.” denotes first- and second-generation methylation measures of aging residualized for chronological age to derive biological age acceleration metrics

We evaluated the cross-tissue similarity of cell composition-corrected MPSs between saliva and blood samples collected from the same individuals. We found that cross-tissue ICCs were moderate for second-generation indices of biological age acceleration, third-generation pace of aging, and MPSs of physiology and cognition (see Fig. 1 and Supplementary Tables 1, 2, and 3) [44]. As illustrated in Fig. 1, PCGrimAge had the highest ICC (0.76, 95% CI = 0.67–0.84), followed by PCPhenoAge (0.72, CI = 0.61–0.81), a measure of cognitive performance (Epigenetic-*g*, 0.69, CI = 0.50–0.89), DunedinPACE (0.68, CI = 0.57–0.79), PCGrimAge Acceleration (0.67, CI = 0.46–0.87), PCPhenoAge Acceleration (0.66, CI = 0.48–0.84), an indicator of chronic inflammation (DNAm-CRP, 0.58, CI = 0.41–0.73), and body mass index (DNAm-BMI, 0.54, CI = 0.40–0.67). The ICC for the CpG-based PhenoAge Acceleration (0.50, CI = 0.26–0.73) was slightly lower, but still moderate, compared to PC-based PhenoAge Acceleration (0.66, CI = 0.48–0.84).

Next, we compared cross-tissue ICCs to previous reports on within-tissue ICCs collected from the same people. Cross-tissue ICCs appear lower than previous within-tissue ICC reports, although comparison is hindered by a lack of 95% CI in previous studies (saliva ICCs range from 0.67 for DNAm-BMI to 0.85 for PCGrimAge, blood ICCs range from 0.73 for DunedinPACE to 0.93 for PCGrimAge) [2, 16, 17, 29, 30]. We note that within-tissue ICCs of PCGrimAge Acceleration, PCPhenoAge Acceleration, and within-blood ICCs of DNAm-BMI and Epigenetic-*g* were not available in previous studies and are therefore not depicted in Fig. 1.

**Fig. 2:**
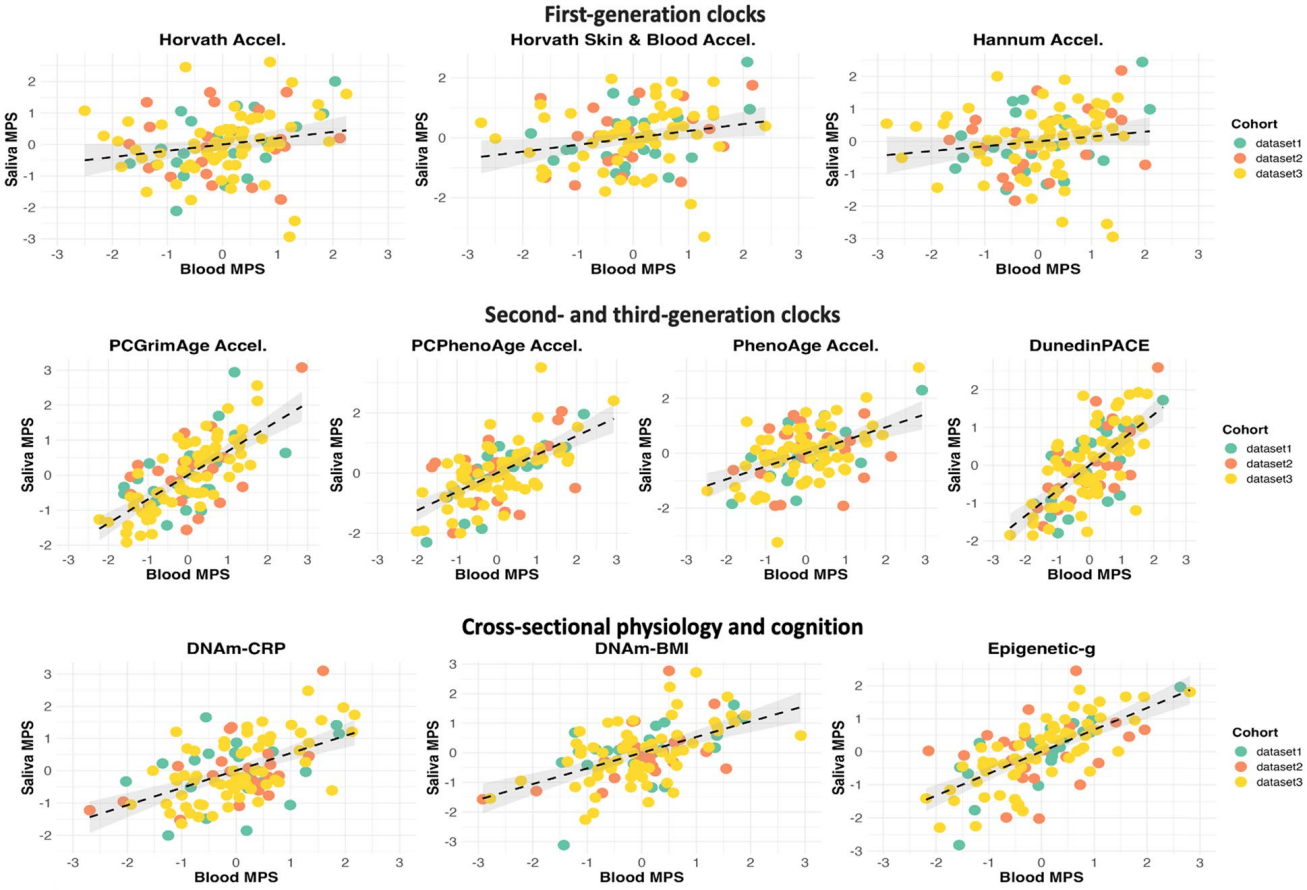
Scatterplot depicting the association between DNA-methylation measures of aging, cross-sectional physiology, and cognition computed in blood and saliva from the same individuals. Each plot shows the methylation profile score (MPS) in blood on the x-axis and the corresponding MPS in saliva on the y-axis. From top left to bottom right, MPSs are Horvath Acceleration, Horvath Skin and Blood Acceleration, Hannum Acceleration, PCGrimAge Acceleration, PCPhenoAge Acceleration, DunedinPACE, DNAm-CRP (a marker of chronic inflammation), DNAm-BMI (body mass index), and Epigenetic-*g* (cognitive performance). The color denotes the three different cohorts: dataset1 (GSE111165, *n* = 21), dataset2 (*GSE130153*, *n* = 22), and dataset3 (*GSE61653, n* = 64). Each plot includes a regression line with shaded confidence intervals, indicating the strength and reliability of the associations. “Accel.” denotes first- and second-generation methylation measures of aging residualized for chronological age to derive biological age acceleration metrics

We further probed whether ICC values significantly varied between datasets. Our meta-analysis suggested that the ICC values for Epigenetic-*g*, Horvath Acceleration, and CpG-based PhenoAge varied significantly between studies as evidenced by heterogeneity test results (*p* = 0.04 for Epigenetic-*g*, *p* = 0.04 for Horvath Acceleration, and *p* = 0.006 for PhenoAge, Supplementary Tables 1 and 3). Figure 2 provides scatterplots depicting the association between MPSs in blood and saliva colored by dataset. See Supplementary Tables 1, 2 and 3 for detailed cross-tissue correlations within and across studies.

Next, we tested whether the statistical method used to estimate cell composition affected the cross-tissue ICCs. We observed that ICC point estimates were generally higher with reference-free correction compared to reference-based correction across all MPSs. However, these differences were not statistically significant, except for PCGrimAge and PCPhenoAge, which showed significantly higher ICC point estimates with reference-free estimation (see Supplementary Table 4).

To facilitate comparison across studies of DNAm measures of aging, we report ICC values for first-generation clocks. Cross-tissue ICC values for first-generation biological age acceleration measures were poor, ranging from 0.19 to 0.25 (see Fig. 1 and Supplementary Table 3). When first-generation clocks were not adjusted for chronological age, the ICC values were moderate, ranging from 0.69 to 0.74. Since Horvath clocks are calibrated on chronological age using DNAm from various tissue types, the ICC values for first-generation clocks were excellent when they were not corrected for cell-type variation or chronological age to derive biological age estimates (*e.g.,* ICC = 0.97 for the Horvath skin and blood clock). Supplementary Table 3 provides ICCs and descriptive statistics for MPSs uncorrected for cell composition.

Lastly, we probed whether ICCs differed by chronological age in a linear regression where saliva MPS was regressed on blood MPS, age, and an interaction term of blood MPS and age. We found that the association of blood and saliva MPSs did not differ by age for any of measures with the exception of PCGrimAge, Horvath, and Horvath Skin and Blood Acceleration. We probed the nature of this interaction by splitting the sample into older and younger individuals based on the overall mean age of 37.12 years. The cross-tissue ICC for GrimAge Acceleration was higher in older participants (ICC = 0.82, CI = 0.68–0.94) compared to younger participants (ICC = 0.49, CI = 0.03–0.94). In contrast, older participants exhibited lower ICCs for Horvath Acceleration (ICC = 0.26, CI = − 0.15–0.68) and Horvath Skin and Blood Acceleration (ICC = − 0.08, CI = − 0.73–0.56) than younger participants, who had ICCs of 0.42 (CI = 0.08–0.76) for Horvath Acceleration and 0.50 (CI = 0.24–0.75) for Horvath Skin and Blood Acceleration.

## Discussion

We meta-analyzed data from three publicly available studies that collected DNAm from both blood and saliva tissues from the same persons to examine the cross-tissue similarity of MPSs of biological aging and cross-sectional physiology and cognition. We focused on MPSs that have previously been found to be sensitive to social determinants of health in both adults and children [45], and note that to-date pediatric studies are substantially more likely to have saliva DNAm (Rezaki, Willems et al., in progress). To enable comparison across metrics and studies, we also report results for first-generation epigenetic clocks developed from analyses of age differences in DNAm.

Our findings suggest that applying DNAm algorithms, originally developed in blood samples, to saliva DNAm and adjusting for cell composition leads to moderate cross-tissue correspondence for second- and third-generation indices of aging, physiology, and cognition, with ICCs ranging from 0.50 to 0.76. In contrast, the cross-tissue ICC values for first-generation biological age acceleration measures were poor, ranging from 0.19 to 0.25. In line with recent recommendations to adjust for cell-type variation [8, 46], our comparison with ICC values uncorrected for cell-type composition suggests that such corrections enhance the reliability of second- and third-generation clocks, and MPSs of physiological measures and cognition. Notably, ICC values for first-generation clocks were excellent when not corrected for cell-type variation or chronological age, as expected due to their calibration on chronological age using DNAm from various tissue types. For most MPSs, the method of cell-type correction, whether reference-based or reference-free, did not significantly impact ICCs.

These cross-tissue ICCs for second- and third-generation indices of aging, physiology, and cognition appear to be lower than previously reported ICCs collected from the same tissue in the same individuals. For example, saliva ICCs range from 0.67 for DNAm-BMI to 0.85 for PCGrimAge, while blood ICCs range from 0.73 for DunedinPACE to 0.93 for PCGrimAge [2, 16, 17, 29, 30]. However, even these within-tissue ICCs are not all classified as “excellent.” Therefore, efforts to increase MPS reliability are needed. For instance, in the development of DunedinPACE, Belsky et al. [20] excluded CpG probes with low test–retest reliability and used elastic-net regression to improve biomarker reliability of the pace of aging.

Given the moderate cross-tissue correspondence, validating blood-based MPSs in saliva by examining their association with corresponding phenotypes is crucial. This task is particularly challenging for second- and third-generation measures of biological aging, which lack a gold-standard metric for validation, especially in younger individuals. However, saliva DNAm measures of physiology and cognition have been explored for phenotypic validation. For instance, saliva DNAm-CRP has shown modest correspondence to serum CRP levels in children and young adults (*r* = 0.27) [47]. Yet, studies in adults suggest that DNAm-CRP shows greater longitudinal stability, potentially offering more stable reflections of cumulative inflammatory burden than traditional serum approaches. This stability could explain the stronger associations with brain outcomes compared to serum CRP [48, 49]. Further, saliva DNAm-BMI has demonstrated bidirectional longitudinal associations with phenotypic BMI across adolescence as well as sensitivity to monozygotic discordance in BMI of 8- to 18-year-old twins [16]. Additionally, saliva Epigenetic-*g* has been shown to correlate significantly with children’s cognitive performance and academic achievement [17]. Thus, these MPSs of physiology and cognition have shown biomarker sensitivity on other metrics using saliva DNAm. Nevertheless, we caution that the moderate cross-tissue ICCs indicate that some signal is likely to be lost.

In contrast to the moderate cross-tissue similarity between saliva and blood, the correlation between buccal and blood tissue for second- and third-generation clocks, as well as Epigenetic-*g*, has been reported to be low to moderate (*r* = 0.25 to *r* = 0.48) [50]. This difference may be due to the fact that blood and saliva samples are both cell mixtures partially composed of the same immune cells (leukocytes) [6]. Although we made statistical adjustments for people’s cell composition, DNAm in immune cells appears to be particularly sensitive to social determinants of health and aging-related inflammation across various tissues [51, 52]. Buccal samples, on the other hand, primarily consists of epithelial cells (80%), rather than leukocytes (20%) [53, 54]. Accordingly, effect size estimates of buccal MPSs with social determinants of health are approximately 50% lower than those reported in previously published analyses of blood MPSs [45, 50]. In contrast, second- and third-generation saliva MPSs of biological aging appear to show similar effect size estimates compared to those of blood, even when assessed in adolescents [13]. Interestingly, buccal versions of second- and third-generation clocks still correlate with self-reported diseases and conditions in adults [55]. This suggests that some of the health-relevant biological aging signals captured by second- and third-generation clocks are shared across tissue types, despite low to moderate reliability of measures.

This study has several limitations. First, the small sample size of 107 individuals may limit the accuracy of ICC estimation, potentially leading to an over- or underestimation of true ICC values. Second, our datasets included very few children, making it difficult to extend our conclusions to younger populations. Third, one of the datasets included individuals with medically intractable epilepsy, a high-risk group that may not be representative of the general population, potentially limiting the broader applicability of our findings. Additionally, the lack of validation studies comparing the associations between saliva-derived MPSs and health-related phenotypes underscore the need for caution in interpreting our findings and applying them in future research.

Finally, there is a lack of studies comparing cross-tissue similarity between other tissues and collection modes, such as dried-blood spots and whole blood. Larger datasets with DNAm collected from multiple tissues at the same time from the same people, including children, are needed.

In conclusion, our findings suggest that applying MPSs of second- and third-generation biological aging and related phenotypes, developed in blood, to saliva results generally in moderate cross-tissue similarity. Our analysis highlights the importance of adjusting for cell composition, as this enhances the reliability of these MPSs in saliva. While the degree of cross-tissue similarity may be adequate for population research settings, it may not be suitable in clinical or commercial applications that require absolute agreement between tissue types to ensure accuracy. Keeping these limitations in mind, saliva MPSs may prove useful to studying the childhood roots of adult health and psychological functioning. This is because saliva is more common than venous blood in large pediatric population cohorts, which are needed to capture social determinants of health. Yet, to continue validating existing and new DNAm algorithms in children, the collection of both blood and saliva DNAm in pediatric samples will be necessary.

## Supplementary Information

Supplementary file 1

Supplementary file 2

## Data Availability

We used three publicly available DNA-methylation datasets from the National Center for Biotechnology Information Gene Expression Omnibus (https://www.ncbi.nlm.nih.gov/geo/) under accession numbers GSE111165, GSE130153, and GSE61653.

## Abbreviations

DNAm: DNA methylation
MPS: Methylation profile score
ICC: Interclass correlation coefficient
BMI: Body mass index
CRP: C-reactive protein
EWAS: Epigenome-wide association study
SNP: Single-nucleotide polymorphisms

## References

[1] Liu L, Van Groen T, Kadish I, et al. Insufficient DNA methylation affects healthy aging and promotes age-related health problems. Clin Epigenetics. 2011;2(2):349–60. 10.1007/s13148-011-0042-6. 22704347 10.1007/s13148-011-0042-6 PMC3365396

[2] Reed RG, Carroll JE, Marsland AL, Manuck SB. DNA methylation-based measures of biological aging and cognitive decline over 16-years: preliminary longitudinal findings in midlife. Aging. 2022. 10.18632/aging.204376. 36374219 10.18632/aging.204376 PMC9792211

[3] Christiansen L, Lenart A, Tan Q, et al. DNA methylation age is associated with mortality in a longitudinal Danish twin study. Aging Cell. 2016;15(1):149–54. 10.1111/acel.12421. 26594032 10.1111/acel.12421 PMC4717264

[4] Saul D, Kosinsky RL. Epigenetics of aging and aging-associated diseases. Int J Mol Sci. 2021;22(1):401. 10.3390/ijms22010401. 33401659 10.3390/ijms22010401 PMC7794926

[5] Hansen TVO, Simonsen MK, Nielsen FC, Hundrup YA. Collection of blood, saliva, and buccal cell samples in a pilot study on the Danish Nurse Cohort: Comparison of the response rate and quality of genomic DNA. Cancer Epidemiol Biomarker Prev. 2007;16(10):2072–6. 10.1158/1055-9965.EPI-07-0611. 10.1158/1055-9965.EPI-07-0611 17932355

[6] Middleton LYM, Dou J, Fisher J, et al. Saliva cell type DNA methylation reference panel for epidemiological studies in children. Epigenetics. 2022;17(2):161–77. 10.1080/15592294.2021.1890874. 33588693 10.1080/15592294.2021.1890874 PMC8865319

[7] Merrill SM, Konwar C, Fatima F, et al. Impact of age-related changes in buccal epithelial cells on pediatric epigenetic biomarker research. Nat Commun. 2025;16(1):609. 10.1038/s41467-025-55909-8. 39800776 10.1038/s41467-025-55909-8 PMC11725590

[8] Guo X, Teschendorff AE. Epigenetic clocks and inflammaging: pitfalls caused by ignoring cell-type heterogeneity. bioRxiv. 2025. 10.1101/2025.01.21.634004.40236252

[9] Teschendorff AE, Horvath S. Epigenetic ageing clocks: statistical methods and emerging computational challenges. Nat Rev Genet. 2025. 10.1038/s41576-024-00807-w. 39806006 10.1038/s41576-024-00807-w

[10] Schrempft S, Stringhini S. Socioeconomic inequalities in the Pace of aging. Aging. 2023;15(6):1706–7. 10.18632/aging.204595. 36917095 10.18632/aging.204595 PMC10085604

[11] Pantell MS, Silveira PP, De Mendonça Filho EJ, et al. Associations between social adversity and biomarkers of inflammation, stress, and aging in children. Pediatr Res. 2024;95(6):1553–63. 10.1038/s41390-023-02992-6. 38233512 10.1038/s41390-023-02992-6 PMC11126389

[12] Raffington L. Utilizing epigenetics to study the shared nature of development and biological aging across the lifespan. npj Sci Learn. 2024. 10.1038/s41539-024-00239-5. 38509146 10.1038/s41539-024-00239-5 PMC10954727

[13] Aikins M, Willems Y, Mitchell C, Goosby B, Raffington L. Linked emergence of racial disparities in mental health and epigenetic biological aging across childhood and adolescence. Mol Psychiatry. 2024. 10.1101/2024.03.26.586786. 10.1038/s41380-025-03010-3 PMC12339396 40205030

[14] Plonski NM, Chen C, Dong Q, et al. Epigenetic age in peripheral blood among children, adolescent, and adult survivors of childhood cancer. JAMA Netw Open. 2023;6(4):e2310325. 10.1001/jamanetworkopen.2023.10325. 37115548 10.1001/jamanetworkopen.2023.10325 PMC10148192

[15] Niccodemi G, Menta G, Turner J, D’Ambrosio C. Pace of aging, family environment and cognitive skills in children and adolescents. SSM - Popul Health. 2022;20:101280. 10.1016/j.ssmph.2022.101280. 36387015 10.1016/j.ssmph.2022.101280 PMC9661391

[16] Raffington L, Schneper L, Mallard T, et al. Salivary epigenetic measures of body mass index and social determinants of health across childhood and adolescence. JAMA Pediatrics. 2023;177(10):1047. 10.1001/jamapediatrics.2023.3017. 37669030 10.1001/jamapediatrics.2023.3017 PMC10481322

[17] Raffington L, Tanksley PT, Sabhlok A, et al. Socially stratified epigenetic profiles are associated with cognitive functioning in children and adolescents. Psychol Sci. 2023;34(2):170–85. 36459657 10.1177/09567976221122760 PMC10068508

[18] Levine ME, Lu AT, Quach A, et al. An epigenetic biomarker of aging for lifespan and healthspan. Aging. 2018;10(4):573–91. 10.18632/aging.101414. 29676998 10.18632/aging.101414 PMC5940111

[19] Lu AT, Quach A, Wilson JG, et al. DNA methylation GrimAge strongly predicts lifespan and healthspan. Aging. 2019;11(2):303–27. 10.18632/aging.101684. 30669119 10.18632/aging.101684 PMC6366976

[20] Belsky DW, Caspi A, Corcoran DL, et al. DunedinPACE, a DNA methylation biomarker of the pace of aging. eLife. 2022. 10.7554/eLife.73420. 35029144 10.7554/eLife.73420 PMC8853656

[21] Ligthart S, Marzi C, Aslibekyan S, et al. DNA methylation signatures of chronic low-grade inflammation are associated with complex diseases. Genome Biol. 2016;17(1):255. 10.1186/s13059-016-1119-5. 27955697 10.1186/s13059-016-1119-5 PMC5151130

[22] Wahl S, Drong A, Lehne B, et al. Epigenome-wide association study of body mass index, and the adverse outcomes of adiposity. Nature. 2017;541(7635):81–6. 10.1038/nature20784. 28002404 10.1038/nature20784 PMC5570525

[23] McCartney DL, Hillary RF, Conole ELS, et al. Blood-based epigenome-wide analyses of cognitive abilities. Genome Biol. 2022;23(1):26. 10.1186/s13059-021-02596-5. 35039062 10.1186/s13059-021-02596-5 PMC8762878

[24] Horvath S. DNA methylation age of human tissues and cell types. Genome Biol. 2013;14(10):3156. 10.1186/gb-2013-14-10-r115. 10.1186/gb-2013-14-10-r115 PMC4015143 24138928

[25] Horvath S, Oshima J, Martin GM, et al. Epigenetic clock for skin and blood cells applied to Hutchinson Gilford Progeria Syndrome and ex vivo studies. Aging. 2018;10(7):1758–75. 10.18632/aging.101508. 30048243 10.18632/aging.101508 PMC6075434

[26] Hannum G, Guinney J, Zhao L, et al. Genome-wide methylation profiles reveal quantitative views of human aging rates. Mol Cell. 2013;49(2):359–67. 10.1016/j.molcel.2012.10.016. 23177740 10.1016/j.molcel.2012.10.016 PMC3780611

[27] Houseman EA, Accomando WP, Koestler DC, et al. DNA methylation arrays as surrogate measures of cell mixture distribution. BMC Bioinform. 2012;13(1):86. 10.1186/1471-2105-13-86. 10.1186/1471-2105-13-86 PMC3532182 22568884

[28] Houseman EA, Kile ML, Christiani DC, Ince TA, Kelsey KT, Marsit CJ. Reference-free deconvolution of DNA methylation data and mediation by cell composition effects. BMC Bioinform. 2016;17(1):259. 10.1186/s12859-016-1140-4. 10.1186/s12859-016-1140-4 PMC4928286 27358049

[29] Schmunk LJ, Call TP, McCartney DL, et al. A novel framework to build saliva-based DNA methylation biomarkers: quantifying systemic chronic inflammation as a case study. Aging Cell. 2023. 10.1101/2023.12.21.572866. 10.1111/acel.14444 PMC11984670 39888134

[30] deSteiguer AJ, Raffington L, Sabhlok A, Tanksley P, Tucker-Drob EM, Harden KP. Stability of DNA-methylation profiles of biological aging in children and adolescents. BioRxiv. 2023. 10.1101/2023.10.30.564766. 37961459 10.1101/2023.10.30.564766 PMC10635005

[31] Braun PR, Han S, Hing B, et al. Genome-wide DNA methylation comparison between live human brain and peripheral tissues within individuals. Transl Psychiatry. 2019;9(1):47. 10.1038/s41398-019-0376-y. 30705257 10.1038/s41398-019-0376-y PMC6355837

[32] Murata Y, Fujii A, Kanata S, et al. Evaluation of the usefulness of saliva for DNA methylation analysis in cohort studies. Neuropsychopharmacol Rep. 2019;39(4):301–5. 10.1002/npr2.12075. 31393092 10.1002/npr2.12075 PMC7292296

[33] Gillespie CF, Bradley B, Mercer K, et al. Trauma exposure and stress-related disorders in inner city primary care patients. Gen Hosp Psychiatry. 2009;31(6):505–14. 10.1016/j.genhosppsych.2009.05.003. 19892208 10.1016/j.genhosppsych.2009.05.003 PMC2785858

[34] Smith AK, Kilaru V, Klengel T, et al. DNA extracted from saliva for methylation studies of psychiatric traits: evidence tissue specificity and relatedness to brain. Am J Med Genet B Neuropsychiatr Genet. 2015;168(1):36–44. 10.1002/ajmg.b.32278. 10.1002/ajmg.b.32278 PMC4610814 25355443

[35] Aryee MJ, Jaffe AE, Corrada-Bravo H, et al. Minfi: a flexible and comprehensive Bioconductor package for the analysis of Infinium DNA methylation microarrays. Bioinformatics. 2014;30(10):1363–9. 10.1093/bioinformatics/btu049. 24478339 10.1093/bioinformatics/btu049 PMC4016708

[36] Assenov Y, Müller F, Lutsik P, Walter J, Lengauer T, Bock C. Comprehensive analysis of DNA methylation data with RnBeads. Nat Methods. 2014;11(11):1138–40. 25262207 10.1038/nmeth.3115 PMC4216143

[37] Barfield RT, Kilaru V, Smith AK, Conneely KN. CpGassoc: an R function for analysis of DNA methylation microarray data. Bioinformatics. 2012;28(9):1280–1. 22451269 10.1093/bioinformatics/bts124 PMC3577110

[38] Teschendorff AE, Marabita F, Lechner M, et al. A beta-mixture quantile normalization method for correcting probe design bias in Illumina Infinium 450 k DNA methylation data. Bioinformatics. 2013;29(2):189–96. 10.1093/bioinformatics/bts680. 23175756 10.1093/bioinformatics/bts680 PMC3546795

[39] Houseman EA, Molitor J, Marsit CJ. Reference-free cell mixture adjustments in analysis of DNA methylation data. Bioinformatics. 2014;30(10):1431–9. 10.1093/bioinformatics/btu029. 24451622 10.1093/bioinformatics/btu029 PMC4016702

[40] Reinius LE, Acevedo N, Joerink M, et al. Differential DNA methylation in purified human blood cells: implications for cell lineage and studies on disease susceptibility. PLoS ONE. 2012;7(7):e41361. 10.1371/journal.pone.0041361. 22848472 10.1371/journal.pone.0041361 PMC3405143

[41] Heiss JA, Just AC. Identifying mislabeled and contaminated DNA methylation microarray data: an extended quality control toolset with examples from GEO. Clin Epigenetics. 2018;10(1):73. 10.1186/s13148-018-0504-1. 29881472 10.1186/s13148-018-0504-1 PMC5984806

[42] Dieckmann L, Cruceanu C, Lahti-Pulkkinen M, et al. Reliability of a novel approach for reference-based cell type estimation in human placental DNA methylation studies. Cell Mol Life Sci. 2022;79(2):115. 10.1007/s00018-021-04091-3. 35113241 10.1007/s00018-021-04091-3 PMC8813756

[43] DerSimonian R, Laird N. Meta-analysis in clinical trials. Control Clin Trials. 1986;7(3):177–88. 10.1016/0197-2456(86)90046-2. 3802833 10.1016/0197-2456(86)90046-2

[44] Koo TK, Li MY. A guideline of selecting and reporting intraclass correlation coefficients for reliability research. J Chiropr Med. 2016;15(2):155–63. 10.1016/j.jcm.2016.02.012. 27330520 10.1016/j.jcm.2016.02.012 PMC4913118

[45] Raffington L, Belsky DW. Integrating DNA methylation measures of biological aging into social determinants of health research. Curr Environ Health Rep. 2022;9(2):196–210. 35181865 10.1007/s40572-022-00338-8

[46] Ming CMH, Meijer M, Merrill SM, et al. Considerations for cell type heterogeneity in pediatric salivary DNA methylation analyses: comparison of reference panels & stratification by estimated cell type proportion. bioRxiv. 2024. 10.1101/2024.11.08.621377. 39763908 10.1101/2024.12.17.628915 PMC11702634

[47] Mrug S, Barker-Kamps M, Orihuela CA, Patki A, Tiwari HK. Childhood neighborhood disadvantage, parenting, and adult health. Am J Prev Med. 2022;63(1):S28–36. 10.1016/j.amepre.2022.01.028. 35725138 10.1016/j.amepre.2022.01.028 PMC9219037

[48] Conole ELS, Stevenson AJ, Muñoz Maniega S, et al. DNA methylation and protein markers of chronic inflammation and their associations with brain and cognitive aging. Neurology. 2021;97(23):e2340–52. 10.1212/WNL.0000000000012997. 34789543 10.1212/WNL.0000000000012997 PMC8665430

[49] Stevenson AJ, McCartney DL, Hillary RF, et al. Characterisation of an inflammation-related epigenetic score and its association with cognitive ability. Clin Epigenetics. 2020. 10.1186/s13148-020-00903-8. 32718350 10.1186/s13148-020-00903-8 PMC7385981

[50] Raffington L, Schwaba T, Aikins M, et al. Associations of socioeconomic disparities with buccal DNA-methylation measures of biological aging. Clin Epigenetics. 2023;15(1):70. 10.1186/s13148-023-01489-7. 37118759 10.1186/s13148-023-01489-7 PMC10148429

[51] Bermick J, Schaller M. Epigenetic regulation of pediatric and neonatal immune responses. Pediatr Res. 2022;91(2):297–327. 10.1038/s41390-021-01630-3. 34239066 10.1038/s41390-021-01630-3

[52] Mavromatis LA, Rosoff DB, Bell AS, Jung J, Wagner J, Lohoff FW. Multi-omic underpinnings of epigenetic aging and human longevity. Nat Commun. 2023;14(1):2236. 10.1038/s41467-023-37729-w. 37076473 10.1038/s41467-023-37729-w PMC10115892

[53] Theda C, Hwang SH, Czajko A, Loke YJ, Leong P, Craig JM. Quantitation of the cellular content of saliva and buccal swab samples. Sci Rep. 2018;8(1):6944. 10.1038/s41598-018-25311-0. 29720614 10.1038/s41598-018-25311-0 PMC5932057

[54] Wong YT, Tayeb MA, Stone TC, et al. A comparison of epithelial cell content of oral samples estimated using cytology and DNA methylation. Epigenetics. 2021;17(3):327–34. 10.1080/15592294.2021.1950977. 34254878 10.1080/15592294.2021.1950977 PMC8920143

[55] Willems YE, deSteiguer A, Tanksley PT, et al. Self-control is associated with health-relevant disparities in buccal DNA-methylation measures of biological aging in older adults. Clin Epigenetics. 2024;16(1):22. 10.1186/s13148-024-01637-7. 38331797 10.1186/s13148-024-01637-7 PMC10854186

[56] Higgins-Chen AT. A computational solution for bolstering reliability of epigenetic clocks: implications for clinical trials and longitudinal tracking. Tech Rep. 2022;2:644–61. 10.1038/s43587-022-00248-2 PMC9586209 36277076

[57] Sugden K, Hannon EJ, Arseneault L, et al. Patterns of reliability: assessing the reproducibility and integrity of DNA methylation measurement. Patterns. 2020;1(2):100014. 10.1016/j.patter.2020.100014. 32885222 10.1016/j.patter.2020.100014 PMC7467214

