# Supplementary material for: Applying blood-derived epigenetic algorithms to saliva: cross-tissue similarity of DNA-methylation indices of aging, physiology, and cognition
Source: Clin Epigenetics. 2025 Apr 23;17:61. doi: 10.1186/s13148-025-01868-2 (PMC12016411; doi:10.1186/s13148-025-01868-2)
Supplement: Supplementary file 1 [file 13148_2025_1868_MOESM1_ESM.docx]

**Applying blood-derived epigenetic algorithms to saliva: Cross-tissue similarity of DNA methylation indices of aging, physiology, and cognition**

Zarandooz, Sepideh & Raffington, Laurel

Supplemental Tables

| **Supplementary Table 1.** Statistics and correlations of second and third generation epigenetic clocks and DNA methylation measures of health and cognition scores in blood and saliva. | | | | | |
| --- | --- | --- | --- | --- | --- |
|  | **Study GSE111165 (n = 21)** | | **Study GSE130153 (n = 22)** | | **Study GSE61653 (n = 64)** |
| ***PCGrimAge Acceleration*** | | | | | |
| Blood | m= 0.0 SD= 3.72 | | m= 0.0 SD= 2.04 | | m= 0.0 SD= 3.19 |
| Saliva | m=0.0 SD= 4.42 | | m= 0.0 SD= 2.49 | | m=0.0 SD= 3.31 |
| Meta-analyzed ICC reference-free cell correction | ICC= 0.67, 95%CI= [0.46, 0.87], *p*<0.001, *H-p*= 0.10 | | | | |
| Meta-analyzed ICC reference-based cell correction | ICC= 0.68, 95%CI= [0.43, 0.94], *p*<0.001, *H-p*= 0.02 | | | | |
| Meta-analyzed ICC unadjusted for cell composition | ICC= 0.49, 95%CI= [0.34, 0.63], *p*<0.001, *H-p*= 0.31 | | | | |
| ICC unadjusted for cell composition | ICC= 0.56, 95%CI= [0.18, 0.80], *p*=0.003 | | ICC= 0.63, 95%CI= [0.29, 0.83], *p*<0.001 | | ICC= 0.40, 95%CI= [0.18, 0.52], *p*<0.001 |
| ICC reference-free cell correction | ICC= 0.52, 95%CI= [0.11, 0.77], *p*=0.007 | | ICC= 0.54, 95%CI= [0.15, 0.78], *p*=0.004 | | ICC= 0.80, 95%CI= [0.68, 0.87], *p*<0.001 |
| ICC reference-based cell correction | ICC= 0.57, 95%CI= [0.19, 0.80], *p*=0.003 | | ICC= 0.51, 95%CI= [0.11, 0.76], *p*=0.007 | | ICC= 0.86, 95%CI= [0.78, 0.91], *p*<0.001 |
| Pearson’s correlation reference-free cell correction | *r*=0.50, 95%CI=[0.09, 0.77], *p*=0.018 | | *r*=0.53, 95%CI=[0.14, 0.78], *p*=0.010 | | *r*=0.79, 95%CI=[0.68, 0.87], *p*<0.001 |
| Pearson’s correlation reference-based cell correction | *r*=0.56, 95%CI=[0.17, 0.80], *p*=0.007 | | *r*=0.5, 95%CI= [0.10, 0.76], *p*=0.017 | | *r*=0.86, 95%CI=[0.78, 0.91], *p*<0.001 |
| ***PCGrimAge*** | | | | | |
| Blood | | m= 49.99 SD= 13.87 | | m= 44.68 SD= 5.22 | m= 57.43 SD= 10.18 |
| Saliva | | m= 59.52 SD= 12.42 | | m= 54.16 SD= 5.70 | m= 66.14 SD= 10.10 |
| Meta-analyzed ICC reference-free cell correction | | ICC= 0.76, 95%CI= [0.67, 0.84], p<0.001, *H-p*= 0.52 | | | |
| Meta-analyzed ICC reference-based cell correction | | ICC= 0.19, 95%CI= [-0.05, 0.43], p=0.13, *H-p*= 0.78 | | | |
| Meta-analyzed ICC unadjusted for cell composition | | ICC= 0.56, 95%CI= [0.31, 0.82], p<0.001, *H-p*= 0.37 | | | |
| ICC unadjusted for cell composition | | ICC= 0.75, 95%CI= [-0.06, 0.93], *p*=0.05 | | ICC= 0.36, 95%CI= [-0.02, 0.76], *p*=0.12 | ICC= 0.71, 95%CI= [-0.03, 0.92], *p*=0.06 |
| ICC reference-free cell correction | | ICC= 0.83, 95%CI= [0.63, 0.93], *p*<0.001 | | ICC= 0.69, 95%CI= [0.39, 0.86], *p*<0.001 | ICC= 0.74, 95%CI= [0.60, 0.83], *p*<0.001 |
| ICC reference-based cell correction | | ICC= 0.17, 95%CI= [-0.29, 0.56], *p*=0.23 | | ICC= 0.09, 95%CI= [-0.36, 0.49], *p*=0.35 | ICC= 0.30, 95%CI= [-0.34, 0.50], *p*=0.008 |
| Pearson’s correlation reference-free cell correction | | *r*=0.82, 95%CI= [0.61, 0.93], *p*<0.001 | | *r*=0.68, 95%CI= [0.37, 0.86], *p*<0.001 | *r*=0.73, 95%CI= [0.60, 0.83], *p*<0.001 |
| Pearson’s correlation reference-based cell correction | | *r*=0.16, 95%CI= [-0.28, 0.55], *p*=0.47 | | *r*=0.08, 95%CI= [-0.35, 0.48], *p*=0.70 | *r*=0.29, 95%CI=[0.05, 0.50], *p*=0.18 |
| ***PCPhenoAge Acceleration*** | | | | | |
| Blood | m= 0.0 SD= 7.93 | | m= 0.0 SD= 4.32 | | m= 0.0 SD= 5.90 |
| Saliva | m= 0.0 SD= 12.54 | | m= 0.0 SD= 4.58 | | m= 0.0 SD= 9.47 |
| Meta-analyzed ICC reference-free cell correction | ICC= 0.66, 95%CI= [0.48, 0.84], *p*<0.001, *H-p*= 0.11 | | | | |
| Meta-analyzed ICC reference-based cell correction | ICC= 0.51, 95%CI= [0.37, 0.65], *p*<0.001, *H-p*= 0.42 | | | | |
| Meta-analyzed ICC unadjusted for cell composition | ICC= 0.22, 95%CI= [0.01, 0.40], *p*=0.01, *H-p*= 0.76 | | | | |
| ICC unadjusted for cell composition | ICC= 0.12, 95%CI= [-0.34, 0.52], *p*=0.05 | | ICC= 0.16, 95%CI= [-0.28, 0.54], *p*=0.12 | | ICC= 0.28, 95%CI= [0.03, 0.49], *p*=0.06 |
| ICC reference-free cell correction | ICC= 0.80, 95%CI= [0.55, 0.91], *p*<0.001 | | ICC= 0.37, 95%CI= [-0.07, 0.68], *p*=0.005 | | ICC= 0.65, 95%CI= [0.48, 0.77], *p*<0.001 |
| ICC reference-based cell correction | ICC= 0.59, 95%CI= [0.21, 0.81], *p*=0.002 | | ICC= 0.27, 95%CI= [-0.18, 0.61], *p*=0.11 | | ICC= 0.53, 95%CI= [0.33, 0.69], *p*<0.001 |
| Pearson’s correlation reference-free cell correction | *r*=0.77, 95%CI= [0.5, 0.9], *p*<0.001 | | *r*=0.33, 95%CI= [-0.1, 0.66], *p*=0.13 | | *r*=0.64, 95%CI= [0.47, 0.76], *p*<0.001 |
| Pearson’s correlation reference-based cell correction | *r*=0.57, 95%CI= [0.19, 0.8], *p*=0.006 | | *r*=0.24, 95%CI= [-0.19, 0.6], *p*=0.27 | | *r*=0.51, 95%CI=[0.3, 0.67], *p*<0.001 |
| ***PCPhenoAge*** | | | | | |
| Blood | | m= 38.20 SD= 20.59 | | m= 32.33 SD= 7.08 | m= 44.66 SD= 12.12 |
| Saliva | | m= 67.67 SD= 18.33 | | m= 57.12 SD= 6.70 | m= 67.84 SD= 15.44 |
| Meta-analyzed ICC reference-free cell correction | | ICC= 0.72, 95%CI= [0.61, 0.81], p<0.001, *H-p*= 0.41 | | | |
| Meta-analyzed ICC reference-based cell correction | | ICC= 0.26, 95%CI= [0.07, 0.43], p=0.004, *H-p*= 0.61 | | | |
| Meta-analyzed ICC unadjusted for cell composition | | ICC= 0.16, 95%CI= [0.001, 0.31], p=0.04, *H-p*= 0.35 | | | |
| ICC unadjusted for cell composition | | ICC= 0.33, 95%CI= [-0.09, 0.70], *p*=0.13 | | ICC= 0.08, 95%CI= [-0.02, 0.34], *p*=0.21 | ICC= 0.31, 95%CI= [-0.07, 0.67], *p*=0.14 |
| ICC reference-free cell correction | | ICC= 0.80, 95%CI= [0.58, 0.91], *p*<0.001 | | ICC= 0.62, 95%CI= [0.27, 0.82], *p*<0.001 | ICC= 0.68, 95%CI= [0.53, 0.79], *p*<0.001 |
| ICC reference-based cell correction | | ICC= 0.36, 95%CI= [-0.08, 0.68], *p*=0.05 | | ICC= 0.08, 95%CI= [-0.37, 0.48], *p*=0.36 | ICC= 0.27, 95%CI= [0.03, 0.49], *p*=0.01 |
| Pearson’s correlation reference-free cell correction | | *r*=0.79, 95%CI= [0.56, 0.91], *p*<0.001 | | *r*=0.61, 95%CI= [0.25, 0.82], *p*=0.002 | *r*=0.68, 95%CI= [0.52, 0.79], *p*<0.001 |
| Pearson’s correlation reference-based cell correction | | *r*=0.35, 95%CI= [-0.09, 0.67], *p*=0.12 | | *r*=0.07, 95%CI= [-0.35, 0.48], *p*=0.72 | *r*=0.27, 95%CI=[0.03, 0.48], *p*=0.02 |
| ***DunedinPACE*** | | | | | |
| Blood | m= 1.07, SD=0.11 | | m= 1.07, SD=0.1 | | m= 1.11, SD=0.13 |
| Saliva | m=1.34, SD=0.157 | | m=1.31, SD=0.17 | | m=1.32, SD=0.18 |
| Meta-analyzed ICC reference-free cell correction | ICC= 0.68, 95%CI= [0.57, 0.79], p<0.001, *H-p*= 0.96 | | | | |
| Meta-analyzed ICC reference-based cell correction | ICC= 0.56, 95%CI= [0.42, 0.69], p<0.001, *H-p*= 0.77 | | | | |
| Meta-analyzed ICC unadjusted for cell composition | ICC= 0.10, 95%CI= [-0.03, 0.23], p=0.14, *H-p*= 0.35 | | | | |
| ICC unadjusted for cell composition | ICC= 0.03 [-0.09, 0.23], *p*=0.36 | | ICC= 0.22 [-0.09, 0.58], *p*=0.16 | | ICC= 0.24 [-0.08, 0.52], *p*=0.09 |
| ICC reference-free cell correction | ICC= 0.65 [0.31, 0.84], *p*<0.001 | | ICC= 0.70 [0.40, 0.86], *p*<0.001 | | ICC= 0.68 [0.54, 0.81], *p*<0.001 |
| ICC reference-based cell correction | ICC= 0.52 [0.11, 0.77], *p*=0.007 | | ICC= 0.64 [0.30, 0.83], *p*<0.001 | | ICC= 0.53 [0.33, 0.69], *p*<0.001 |
| Pearson’s correlation reference-free cell correction | *r*= 0.60 [0.23, 0.82], *p*=0.004 | | *r*= 0.69 [0.38, 0.86], *p*=0.0003 | | *r*= 0.67 [0.51, 0.78], *p*<0.001 |
| Pearson’s correlation reference-based cell correction | *r*= 0.39 [-0.04, 0.7], *p*=0.08 | | *r*=0.62 [0.28, 0.83], *p*=0.001 | | *r*=0.52 [0.32, 0.68], *p*<0.001 |
| **C-reactive protein (CRP)** | | | | | |
| Blood | m= 0.005, SD=0.01 | | m= 0.01, SD=0.01 | | m=0.02, SD=0.01 |
| Saliva | m=0.06, SD=0.04 | | m=0.02, SD=0.02 | | m=0.03, SD=0.02 |
| Meta-analyzed ICC reference-free cell correction | ICC= 0.58, 95%CI= [0.43, 0.73], p<0.001, *H-p*= 0.29 | | | | |
| Meta-analyzed ICC reference-based cell correction | ICC= 0.54, 95%CI= [0.40, 0.68], p<0.001, *H-p*= 0.72 | | | | |
| Meta-analyzed ICC unadjusted for cell composition | ICC= 0.20, 95%CI= [-0.12, 0.52], p=0.21, *H-p*= 0.009 | | | | |
| ICC unadjusted for cell composition | ICC= -0.10, 95%CI= [-0.27, 0.19], *p*=0.80 | | ICC= 0.48, 95%CI= [0.08, 0.75], *p*=0.009 | | ICC= 0.27, 95%CI= [0.03, 0.47], *p*=0.01 |
| ICC reference-free cell correction | ICC= 0.32, 95%CI= [-0.14, 0.65], *p*=0.084 | | ICC= 0.54, 95%CI= [0.15, 0.78], *p*=0.004 | | ICC= 0.64, 95%CI= [0.47, 0.77], *p*<0.001 |
| ICC reference-based cell correction | ICC= 0.40, 95%CI= [-0.04, 0.70], *p*=0.084 | | ICC= 0.58, 95%CI= [0.12, 0.77], *p*=0.007 | | ICC= 0.56, 95%CI= [0.37, 0.71], *p*<0.001 |
| Pearson’s correlation reference-free cell correction | *r*=0.28, 95%CI= [-0.17, 0.8  63], *p*=0.21 | | *r*=0.53, 95%CI= [0.14, 0.77], *p*=0.01 | | *r*=0.64, 95%CI=[0.47, 0.76], *p*<0.001 |
| Pearson’s correlation reference-based cell correction | *r*=0.4, 95%CI= [-0.03, 0.7], *p*=0.07 | | *r*=0.5, 95%CI= [0.1, 0.76], *p*=0.01 | | *r*=0.56, 95%CI=[0.36, 0.7], *p*<0.001 |
| **Body mass index (BMI)** | | | | | |
| Blood | m= 593.13, SD=44.46 | | m= 512.66, SD=29.45 | | m=536.586, SD=41.26 |
| Saliva | m=594.6, SD=25.39 | | m=499.33, SD=33.01 | | m=525.09, SD=47.94 |
| Meta-analyzed ICC reference-free cell correction | ICC= 0.54, 95%CI= [0.40, 0.67], *p*<0.001, *H-p*= 0.99 | | | | |
| Meta-analyzed ICC reference-based cell correction | ICC= 0.59, 95%CI= [0.45, 0.74], *p*<0.001, *H-p*= 0.31 | | | | |
| Meta-analyzed ICC unadjusted for cell composition | ICC= 0.59, 95%CI= [0.43, 0.74], p<0.001, *H-p*= 0.29 | | | | |
| ICC unadjusted for cell composition | ICC= 0.39, 95%CI= [-0.05, 0.70], *p*=0.03 | | ICC= 0.75, 95%CI= [0.35, 0.90], *p*<0.001 | | ICC= 0.57, 95%CI= [0.38, 0.72], *p*<0.001 |
| ICC reference-free cell correction | ICC= 0.53, 95%CI= [0.13, 0.78], *p*=0.006 | | ICC= 0.54, 95%CI= [0.15, 0.78], *p*=0.004 | | ICC= 0.54, 95%CI= [0.33, 0.69], *p*<0.001 |
| ICC reference-based cell correction | ICC= 0.33, 95%CI= [-0.13, 0.66], *p*=0.077 | | ICC= 0.69, 95%CI= [0.39, 0.86], *p*<0.001 | | ICC= 0.60, 95%CI= [0.42, 0.74], *p*<0.001 |
| Pearson’s correlation reference-free cell correction | *r*=0.47, 95%CI= [0.05, 0.75], *p*=0.03 | | *r*=0.53, 95%CI= [-0.03, 0.7], *p*<0.001 | | *r*=0.53, 95%CI= [0.33, 0.68], *p*<0.001 |
| Pearson’s correlation reference-based cell correction | *r*=0.52, 95%CI= [0.11, 0.77], *p*=0.01 | | *r*=0.68, 95%CI= [0.36, 0.85], *p*=0.01 | | *r*=0.6, 95%CI= [0.41, 0.73], *p*<0.001 |
| ***Epigenetic-g*** | | | | | |
| Blood | m= 0.0, SD=0.32 | | m= 0.0, SD=0.1 | | m=0.0, SD=0.13 |
| Saliva | m=0.0, SD=0.24 | | m=0.0, SD=0.07 | | m=0.0, SD=0.13 |
| Meta-analyzed ICC reference-free cell correction | ICC= 0.69, 95%CI= [0.50, 0.89], *p*<0.001, *H-p*= 0.04 | | | | |
| Meta-analyzed ICC reference-based cell correction | ICC= 0.52, 95%CI= [0.20, 0.85], p<0.001, *H-p*= 0.009 | | | | |
| Meta-analyzed ICC unadjusted for cell composition | ICC= 0.55, 95%CI= [0.22, 0.88], p=0.001, *H-p*= 0.001 | | | | |
| ICC unadjusted for cell composition | ICC= 0.71, 95%CI= [0.41, 0.87], *p*<0.001 | | ICC= -0.03, 95%CI= [-0.47, 0.39], *p*=0.56 | | ICC= 0.77, 95%CI= [0.64, 0.85], *p*<0.001 |
| ICC reference-free cell correction | ICC= 0.80, 95%CI= [0.57, 0.91], *p*<0.001 | | ICC= 0.25, 95%CI= [-0.20, 0.60], *p*=0.13 | | ICC= 0.76, 95%CI= [0.64, 0.85], *p*<0.001 |
| ICC reference-based cell correction | ICC= 0.63, 95%CI= [0.28, 0.83], *p*=0.001 | | ICC= 0.04, 95%CI= [-0.40, 0.46], *p*=0.42 | | ICC= 0.73, 95%CI= [0.59, 0.83], *p*<0.001 |
| Pearson’s correlation reference-free cell correction | *r*=0.82, 95%CI=[0.6, 0.92], *p*<0.001 | | | *r*=0.24, 95%CI=[-0.19, 0.86], *p*=0.27 | *r*=0.76, 95%CI=[0.63, 0.84], *p*<0.001 |
| Pearson’s correlation reference-based cell correction | *r*=0.57, 95%CI= [0.19, 0.8], *p*=0.006 | | *r*=0.04, 95%CI= [-0.38, 0.45], *p*=0.85 | | *r*=0.72, 95%CI= [0.58, 0.82], *p*<0.001 |
| *Note:* This table summarizes the means and standard deviations of cell composition unadjusted scores (Acceleration measures are residualized on age but unscaled), along with the cross-tissue Pearson and intraclass correlation coefficients (ICCs) for methylation profile scores derived from blood and saliva samples across three datasets. Correlation test P-values and 95% confidence intervals are included. The scores have been adjusted (residualized) for cell composition estimation utilizing both reference-free and reference-based methods, in addition to uncorrected cell-composition scores. Furthermore, the table presents meta-analyzed ICCs between the two tissue types. | | | | | |

| **Supplementary Table 2.** CpG-based PhenoAge methylation scores in blood and saliva: statistics and correlations | | | | | |
| --- | --- | --- | --- | --- | --- |
|  | | **Study GSE111165 (n = 21)** | **Study GSE130153 (n = 22)** | **Study GSE61653 (n = 64)** | |
| ***CpG-based PhenoAge*** | | | | | |
| Blood | m = 11.90, SD= 19.82 | | m = 37.17, SD= 8.48 | | m = 37.12, SD= 13.00 |
| Saliva | m = 20.49, SD= 16.82 | | m = 47.69, SD= 7.80 | | m = 47.84, SD= 14.51 |
| Meta-analyzed ICC reference-free cell correction (Acceleration) | ICC= 0.50, 95%CI= [0.26, 0.73], p= 0.09, *H-P=0.09* | | | | |
| Meta-analyzed ICC reference-free cell correction | ICC= 0.63, 95%CI= [0.37, 0.88], p= 0.006, *H-P=0.006* | | | | |
| Meta-analyzed ICC reference-based cell correction (Acceleration) | ICC= 0.41, 95%CI= [0.19, 0.63], p= 0.19, *H-P=0.19* | | | | |
| Meta-analyzed ICC reference-based cell correction | ICC= 0.71, 95%CI= [0.53, 0.89], p= 0.07, *H-P=0.007* | | | | |
| Meta-analyzed ICC unadjusted for cell composition | ICC= 0.52, 95%CI= [0.13, 0.91], p=0.008, *H-p*= 0.001 | | | | |
| ICC unadjusted for cell composition | ICC= 0.83, 95%CI= [0.13, 0.95], *p*=0.01 | | ICC= 0.20, 95%CI= [-0.10, 0.53], *p*=0.12 | | ICC= 0.61, 95%CI= [-0.03, 0.85], *p*=0.03 |
| ICC reference-free cell correction (Acceleration) | ICC= 0.66, 95%CI= [0.33, 0.85], *p*<0.001 | | ICC= 0.13, 95%CI= [-0.33, 0.51], *p*=0.30 | | ICC= 0.54, 95%CI= [0.34, 0.70], *p*<0.001 |
| ICC reference-free cell correction | ICC = 0.84, 95%CI= [0.64, 0.93], *p*<0.001 | | ICC = 0.16, 95%CI= [-0.30, 0.54], *p*=0.24 | | ICC = 0.66, 95%CI= [0.50, 0.78], *p*<0.001 |
| ICC reference-based cell correction (Acceleration) | ICC= 0.57, 95%CI= [0.19, 0.80], *p*=0.003 | | ICC= 0.09, 95%CI= [-0.36, 0.50], *p*=0.34 | | ICC= 0.44, 95%CI= [0.21, 0.61], *p*<0.001 |
| ICC reference-based cell correction | | ICC = 0.78, 95%CI= [0.52, 0.90], *p*<0.001 | ICC =0.33, 95%CI= [-0.11, 0.66], *p*=0.07 | ICC =0.79, 95% CI= [0.68, 0.87], *p*<0.001 | |
| *Note:* This table summarizes the means and standard deviations of cell composition unadjusted scores, along with the cross-tissue intraclass correlation coefficients (ICCs) for CpG-based PhenoAge methylation profile scores derived from blood and saliva samples across three datasets. Correlation test P-values and 95% confidence intervals are included. The scores have been adjusted (residualized) for cell composition estimation utilizing both reference-free and reference-based methods, in addition to uncorrected cell-composition scores. Furthermore, the table presents meta-analyzed ICCs between the two tissue types. | | | | | |

| ***Supplementary Table 3.*** *First generation clocks methylation scores in blood and saliva: statistics and correlations* | | | | | | | | |
| --- | --- | --- | --- | --- | --- | --- | --- | --- |
|  | | **Study GSE111165 (n = 21)** | | **Study GSE130153 (n = 22)** | | **Study GSE61653 (n = 64)** | | |
| ***Horvath*** | | | | | | | | |
| Blood | m = 41.50, SD= 17.31 | | | m = 32.15, SD= 6.48 | | | m = 40.02, SD= 11.57 | |
| Saliva | m = 36.16, SD= 16.70 | | | m = 30.65, SD= 6.76 | | | m = 40.90, SD= 10.79 | |
| Meta-analyzed ICC reference-free cell correction (Acceleration) | ICC= 0.25, 95%CI= [-0.04, 0.55], p=0.09, *H-P=0.04* | | | | | | | |
| Meta-analyzed ICC reference-free cell correction | ICC= 0.69, 95%CI= [0.50, 0.88], p<0.001, *H-P=0.04* | | | | | | | |
| Meta-analyzed ICC reference-based cell correction (Acceleration) | ICC= 0.37, 95%CI= [0.21, 0.54], p<0.001, *H-P=0.36* | | | | | | | |
| Meta-analyzed ICC reference-based cell correction | ICC= 0.80, 95%CI= [0.74, 0.87], p<0.001, *H-P=0.43* | | | | | | | |
| Meta-analyzed ICC unadjusted for cell composition | ICC= 0.90, 95%CI= [0.86, 0.94], p<0.001, *H-p*= 0.56 | | | | | | | |
| ICC unadjusted for cell composition | ICC= 0.90, 95%CI= [0.50, 0.97], *p*<0.001 | | | ICC= 0.82, 95%CI= [0.60, 0.92], *p*<0.001 | | | ICC= 0.91, 95%CI= [0.86, 0.94], *p*<0.001 | |
| ICC reference-free cell correction (Acceleration) | ICC= 0.55, 95%CI= [0.15, 0.78], *p*=0.005 | | | ICC= 0.02, 95%CI= [-0.42, 0.44], *p*=0.46 | | | ICC= 0.15, 95%CI= [-0.09, 0.38], *p*=0.11 | |
| ICC reference-free cell correction | ICC= 0.85, 95%CI= [0.66, 0.94], *p*<0.001 | | | ICC= 0.52, 95%CI= [0.13, 0.77], *p*=0.006 | | | ICC= 0.63, 95%CI= [0.45, 0.75], *p*<0.001 | |
| ICC reference-based cell correction (Acceleration) | ICC= 0.09, 95%CI= [-0.36, 0.50], *p*=0.34 | | | ICC= 0.42, 95%CI= [0.002, 0.71], *p*=0.02 | | | ICC= 0.43, 95%CI= [0.20, 0.60], *p*<0.001 | |
| ICC reference-based cell correction | ICC= 0.69, 95%CI= [0.38, 0.86], *p*<0.001 | | | ICC= 0.74, 95%CI= [0.47, 0.88], *p*<0.001 | | | ICC= 0.83, 95%CI= [0.74, 0.89], *p*<0.001 | |
| ***Horvath Skin and Blood*** | | | | | | | | |
| Blood | | | m = 35.05, SD= 18.45 | | m = 34.24, SD= 7.11 | | | m = 42.97, SD= 12.12 |
| Saliva | | | m = 34.72, SD= 18.92 | | m = 31.04, SD= 6.43 | | | m = 40.83, SD= 11.70 |
| Meta-analyzed ICC reference-free cell correction (Acceleration) | | | ICC= 0.25, 95%CI= [0.07, 0.42], *p*=0.006, *H-P=0.49* | | | | | |
| Meta-analyzed ICC reference-free cell correction | | | ICC= 0.74, 95%CI= [0.64, 0.84], *p*<0.001, *H-P=0.30* | | | | | |
| Meta-analyzed ICC reference-based cell correction (Acceleration) | | | ICC= 0.30, 95%CI= [0.13, 0.47], *p*<0.001, *H-P=0.44* | | | | | |
| Meta-analyzed ICC reference-based cell correction | | | ICC= 0.83, 95%CI= [0.76, 0.89], *p*<0.001, *H-P=0.73* | | | | | |
| Meta-analyzed ICC unadjusted for cell composition | | | ICC= 0.97, 95%CI= [0.86, 1.0], *p*<0.001, *H-p*= 0.14 | | | | | |
| ICC unadjusted for cell composition | | | ICC= 0.99, 95%CI= [0.98, 0.99], *p*<0.001 | | ICC= 0.79, 95%CI= [0.23, 0.93], *p*=0.005 | | | ICC= 0.94, 95%CI= [0.85, 0.97], *p*<0.001 |
| ICC reference-free cell correction (Acceleration) | | | ICC = 0.42, 95%CI= [-0.02, 0.71], *p*=0.03 | | ICC = 0.29, 95%CI= [-0.15, 0.63], *p*=0.09 | | | ICC = 0.16, 95%CI= [-0.09, 0.39], *p*=0.10 |
| ICC reference-free cell correction | | | ICC= 0.83, 95%CI= [0.63, 0.92], *p*<0.001 | | ICC= 0.69, 95%CI= [0.39, 0.86], *p*<0.001 | | | ICC= 0.68, 95%CI= [0.51, 0.79], *p*<0.001 |
| ICC reference-based cell correction (Acceleration) | | | ICC = 0.36, 95%CI= [-0.08, 0.68], *p*=0.05 | | ICC = 0.47, 95%CI= [0.05, 0.74], *p*=0.01 | | | ICC = 0.21, 95%CI= [-0.04, 0.41], *p*=0.05 |
| ICC reference-based cell correction | | | ICC= 0.77, 95%CI= [0.52, 0.90], *p*<0.001 | | ICC= 0.86, 95%CI= [0.70, 0.94], *p*<0.001 | | | ICC= 0.83, 95%CI= [0.73, 0.89], *p*<0.001 |
| ***Hannum*** | | | | | | | | |
| Blood | | | m = 30.80, SD= 14.38 | | m = 42.68, SD= 7.09 | | m = 46.69, SD= 12.16 | |
| Saliva | | | m = 41.28, SD= 13.13 | | m = 49.25, SD= 5.09 | | m = 55.07, SD= 12.46 | |
| Meta-analyzed ICC reference-free cell correction (Acceleration) | | | ICC= 0.19, 95%CI= [-0.03, 0.42], *p*=0.09, *H-P=0.23* | | | | | |
| Meta-analyzed ICC reference-free cell correction | | | ICC= 0.70, 95%CI= [0.57, 0.83], *p*<0.001, *H-P=0.19* | | | | | |
| Meta-analyzed ICC reference-based cell correction (Acceleration) | | | ICC= 0.20, 95%CI= [0.02, 0.38], *p*=0.02, *H-P=0.89* | | | | | |
| Meta-analyzed ICC reference-based cell correction | | | ICC= 0.79, 95%CI= [0.71, 0.86], *p*<0.001, *H-P=0.88* | | | | | |
| Meta-analyzed ICC unadjusted for cell composition | | | ICC= 0.60, 95%CI= [0.32, 0.87], *p*<0.001, *H-p*= 0.67 | | | | | |
| ICC unadjusted for cell composition | | | ICC= 0.67, 95%CI= [-0.07, 0.99], *p*=0.04 | | ICC= 0.45, 95%CI= [-0.10, 0.78], *p*=0.07 | | ICC= 0.73, 95%CI= [-0.05, 0.91], *p*=0.03 | |
| ICC reference-free cell correction (Acceleration) | | | ICC = 0.38, 95%CI= [-0.06, 0.69], *p*=0.04 | | ICC = 0.30, 95%CI= [-0.14, 0.64], *p*=0.09 | | ICC = 0.03, 95%CI= [-0.21, 0.27], *p*=0.40 | |
| ICC reference-free cell correction | | | ICC= 0.81, 95%CI= [0.60, 0.92], *p*<0.001 | | ICC= 0.70, 95%CI= [0.40, 0.86], *p*<0.001 | | ICC= 0.60, 95%CI= [0.42, 0.74], *p*<0.001 | |
| ICC reference-based cell correction (Acceleration) | | | ICC = 0.26, 95%CI= [-0.20, 0.62], *p*=0.13 | | ICC = 0.12, 95%CI= [-0.33, 0.51], *p*=0.30 | | ICC = 0.21, 95%CI= [-0.03, 0.43], *p*=0.05 | |
| ICC reference-based cell correction | | | ICC= 0.75, 95%CI= [0.47, 0.89], *p*<0.001 | | ICC= 0.77, 95%CI= [0.52, 0.89], *p*<0.001 | | ICC= 0.80, 95%CI= [0.70, 0.87], *p*<0.001 | |
| ICC unadjusted for cell composition | | | ICC = 0.81, 95%CI= [0.58, 0.92], *p*<0.001 | | ICC = 0.69, 95%CI= [0.38, 0.86], *p*<0.001 | | ICC = 0.60, 95%CI= [0.41, 0.74], *p*<0.001 | |
| ICC reference-free cell correction (Acceleration) | | | ICC = 0.26, 95%CI= [-0.19, 0.62], *p*=0.25 | | ICC =0.09, 95%CI= [-0.34, 0.49], *p*=0.68 | | ICC =0.13, 95% CI= [-0.11, 0.37], *p*=0.28 | |
| *Note:* This table summarizes the means and standard deviations of cell composition unadjusted scores, along with the cross-tissue intraclass correlation coefficients (ICCs) for methylation profile scores derived from blood and saliva samples across three datasets. Correlation test P-values and 95% confidence intervals are included. The scores have been adjusted (residualized) for cell composition estimation utilizing both reference-free and reference-based methods, in addition to uncorrected cell-composition scores. Furthermore, the table presents meta-analyzed ICCs between the two tissue types. | | | | | | | | |

| **Supplementary Table 4**. Meta-regression results for the effect of cell correction methods on the interclass correlation coefficients (ICCs). | | | |
| --- | --- | --- | --- |
|  | ***Method Estimate (b)*** | ***95% CI*** | ***p-value*** |
| ***First-generation clocks*** | | | |
| Horvath Acc. | -0.09 | -0.44, 0.25 | 0.58 |
| Horvath | -0.06 | -0.27, 0.13 | 0.49 |
| Horvath Skin and Blood Acc. | -0.05 | -0.29, 0.19 | 0.67 |
| Horvath Skin and Blood | -0.08 | -0.20, 0.02 | 0.11 |
| Hannum Accel. | -0.03 | -0.29, 0.22 | 0.78 |
| Hannum | -0.08 | -0.21, 0.04 | 0.18 |
| ***Second-generation clocks*** | | | |
| PCGrimAge Accel. | -0.03 | -0.37, 0.31 | 0.85 |
| PCGrimAge | 0.46 | 0.20, 0.72 | <.0001* |
| PCPhenoAge Accel. | 0.16 | -0.05, 0.38 | 0.13 |
| PCPhenoAge | 0.46 | 0.25, 0.65 | <.0001* |
| PhenoAge Accel. | 0.09 | -0.23, 0.41 | 0.57 |
| PhenoAge | -0.05 | -0.36, 0.26 | 0.74 |
| ***Third-generation clocks*** | | | |
| DunedinPACE | 0.12 | -0.05, 0.29 | 0.17 |
| ***Cross-sectional physiology*** | | | |
| CRP | *-0.06* | -0.25, 0.13 | 0.52 |
| BMI | *0.06* | -0.13, 0.24 | 0.56 |
| Epigenetic-*g* | 0.12 | -0.23, 0.48 | 0.50 |
| *Note:*  The table presents the results of a mixed effects meta-regression assessing the effect of cell composition estimation methods (reference-based vs. reference-free) on interclass correlation coefficients (ICCs) for different DNA methylation profile scores across tissues. The estimates represent the effect of using the reference-free method compared to the reference-based method, with corresponding 95% confidence intervals and p-values. | | | |

| **Supplementary Table 4.** Summary of linear regression analyses for cell-corrected methylation scores interacting with age in all samples. | | | | | |
| --- | --- | --- | --- | --- | --- |
|  | ***blood MPS × age (b)*** | ***SE*** | ***P-value*** | ***R-squared*** | ***Adjusted R-squared*** |
| ***First-generation clocks*** | | | | | |
| Horvath Accel. | -0.0143 | 0.0070 | 0.0450 * | 0.077 | 0.0501 |
| Skin Horvath Accel. | -0.0143 | 0.0067 | 0.03674 * | 0.0925 | 0.0660 |
| Hannum Accel. | -0.0113 | 0.0067 | 0.0950 | 0.0484 | 0.0207 |
| ***Second-generation clocks*** | | | | | |
| PCGrimAge Accel. | 0.0141 | 0.0056 | 0.0141* | 0.5006 | 0.4861 |
| PCPhenoAge Accel | 0.0046 | 0.0064 | 0.4790 | 0.3826 | 0.3647 |
| PhenoAge Accel. | -0.0098 | 0.0068 | 0.1578 | 0.24 | 0.2178 |
| ***Third-generation clocks*** | | | | | |
| DunedinPACE | -0.0047 | 0.0055 | 0.3924 | 0.46 | 0.44 |
| ***Cross-sectional physiology*** | | | | | |
| CRP | 0.0026 | 0.0059 | 0.6699 | 0.3147 | 0.2947 |
| BMI | 0.0073 | 0.0064 | 0.2593 | 0.2977 | 0.2773 |
| Epigenetic-*g* | -0.0023 | 0.0051 | 0.6508 | 0.4548 | 0.4389 |
