# Supplementary material for: Applying blood-derived epigenetic algorithms to saliva: cross-tissue similarity of DNA-methylation indices of aging, physiology, and cognition
Source: Clin Epigenetics. 2025 Apr 23;17:61. doi: 10.1186/s13148-025-01868-2 (PMC12016411; doi:10.1186/s13148-025-01868-2)
Supplement: Supplementary file 2 [file 13148_2025_1868_MOESM2_ESM.docx]

**Applying blood-derived epigenetic algorithms to saliva: Cross-tissue similarity of DNA methylation indices of aging, physiology, and cognition**

Zarandooz, Sepideh & Raffington, Laurel

Supplemental Figures

| **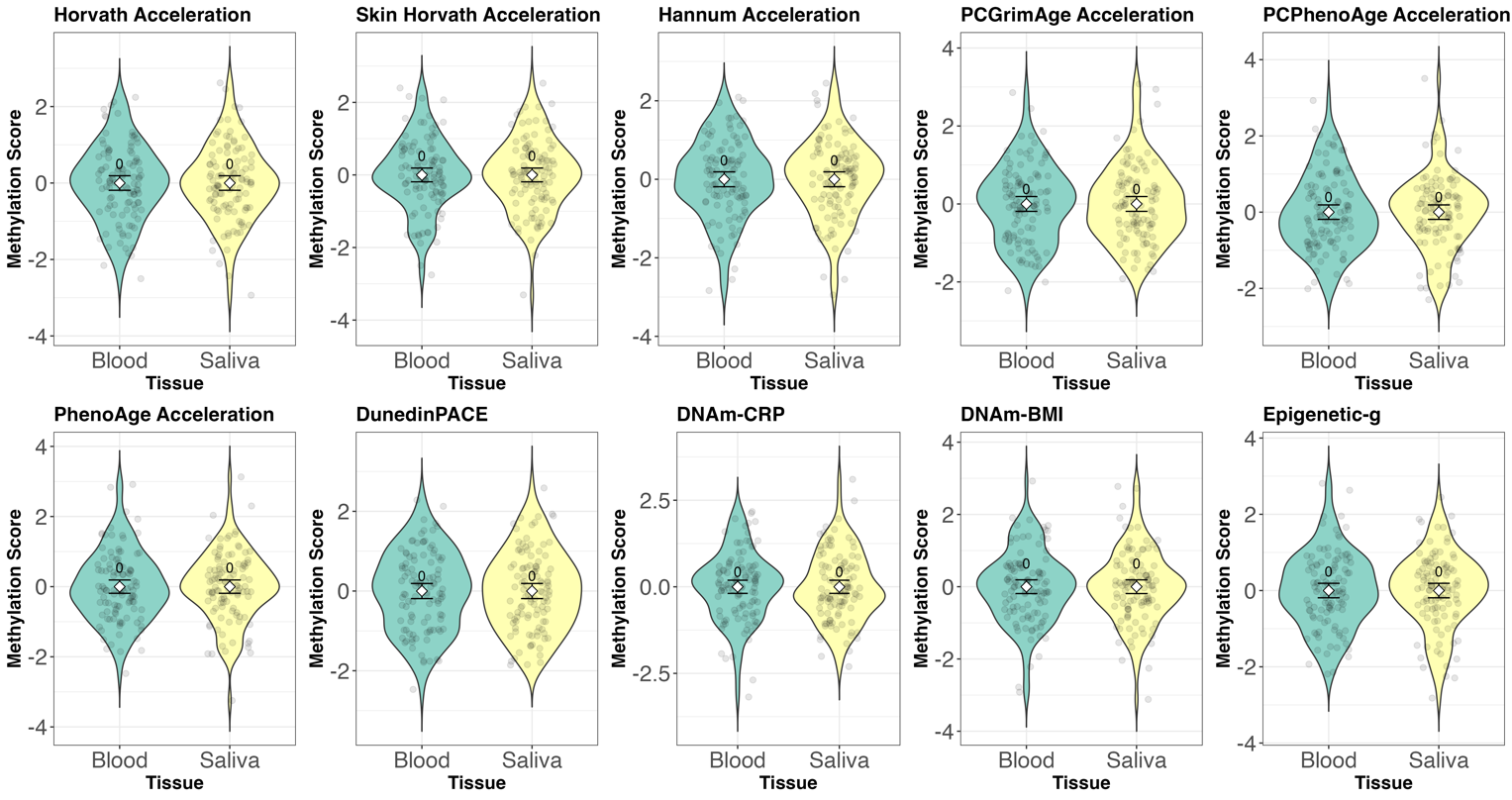** |
| --- |
| **Supplementary Figure 1. Cell-corrected DNA methylation profile scores across all datasets (n=107).** This figure shows the distributions and means of the reference-free cell composition corrected methylation profile scores. "Skin Horvath" denotes the Horvath Skin and Blood epigenetic clock. |

| **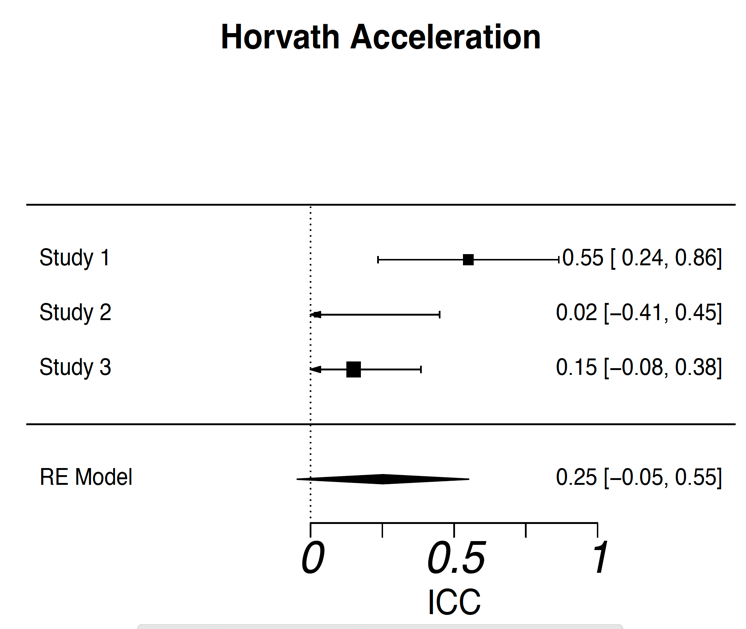** | **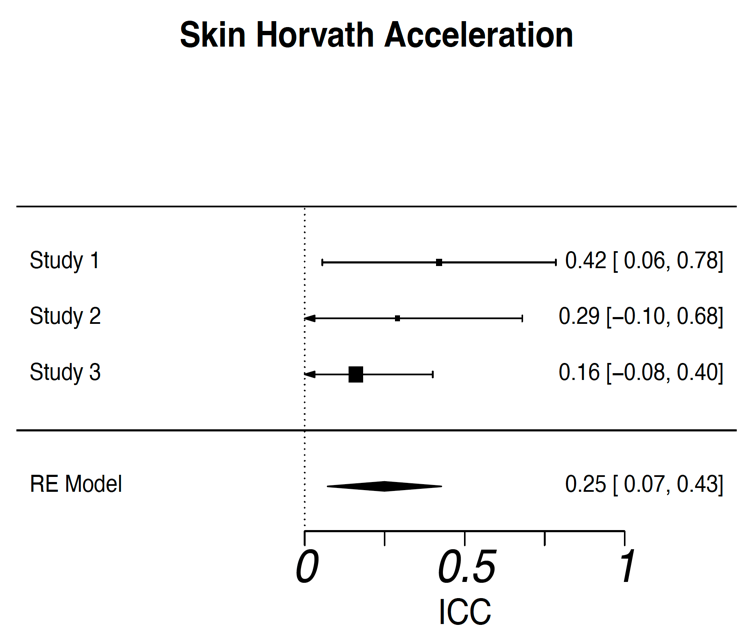** |
| --- | --- |
| 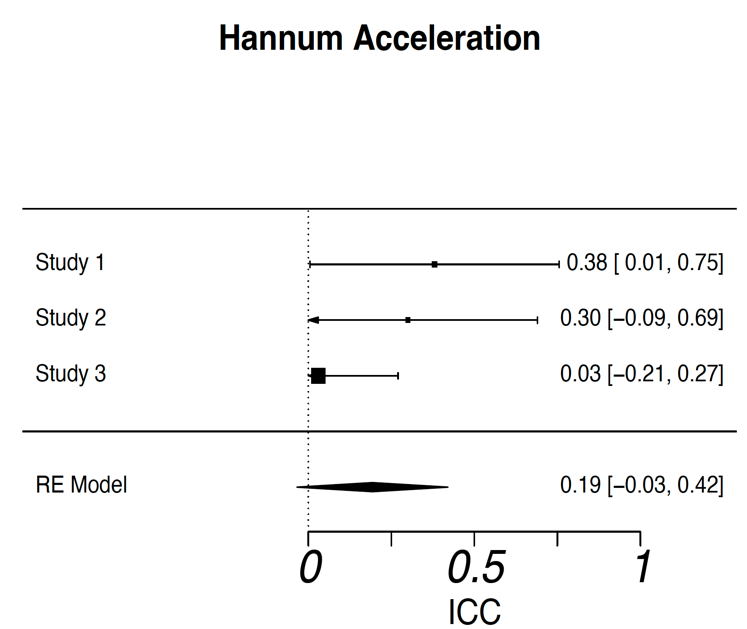 | **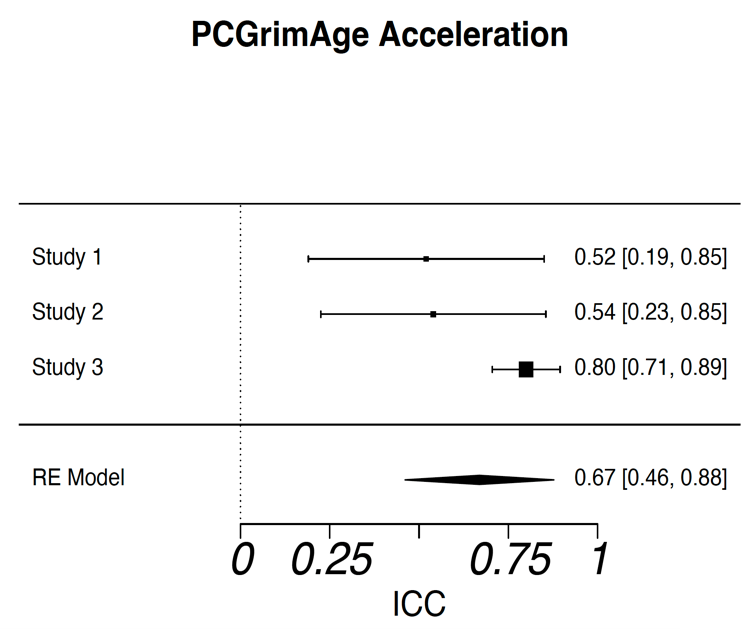** |
| **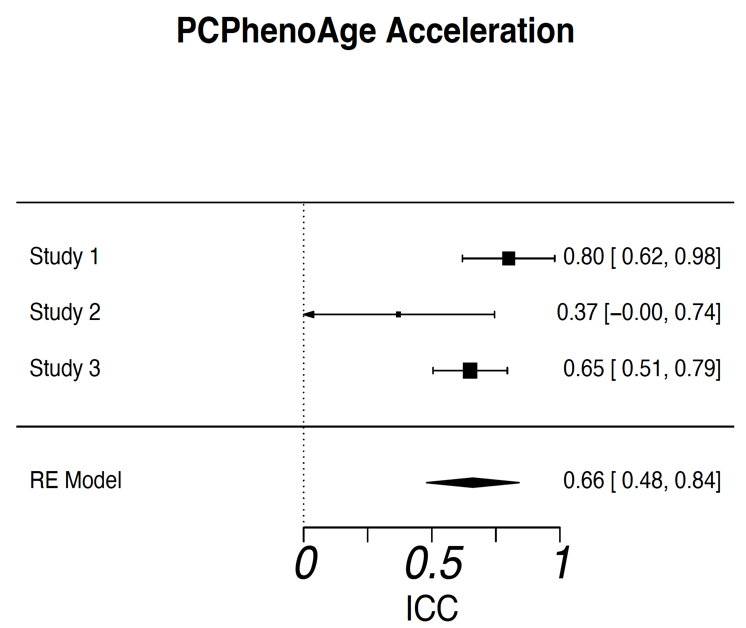** | **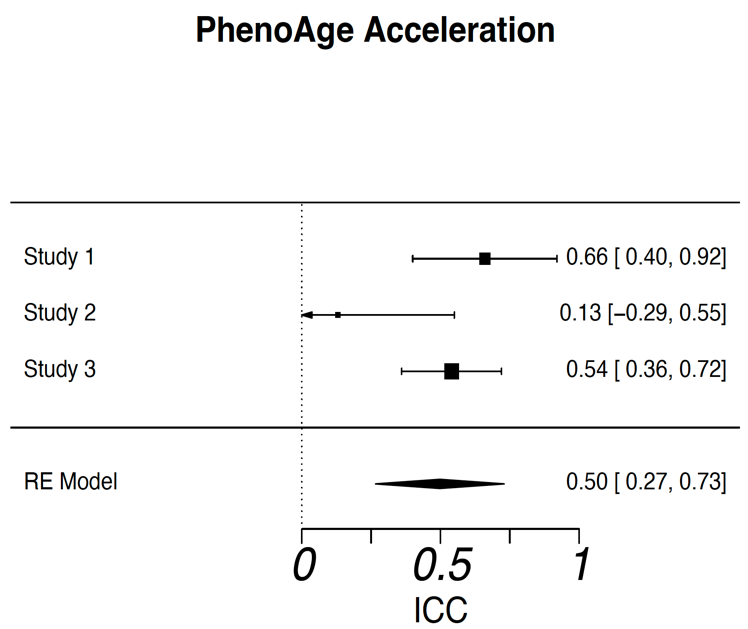** |
| 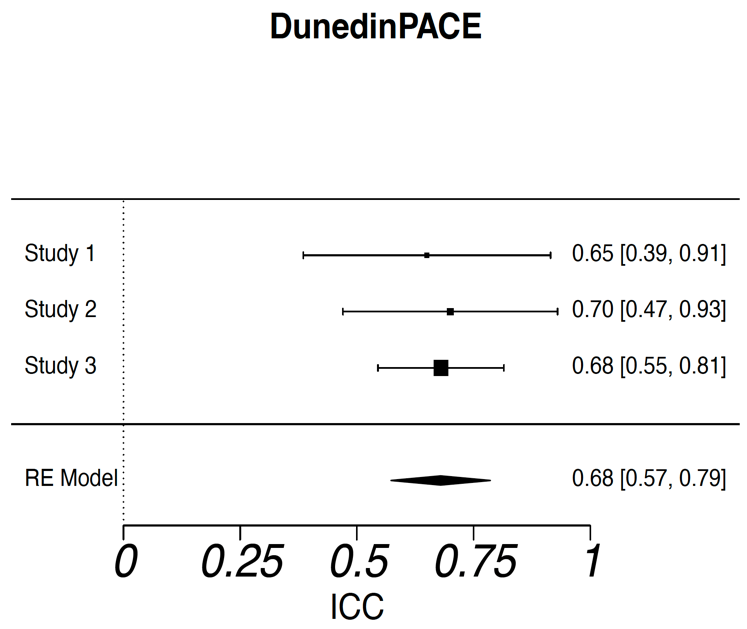 | 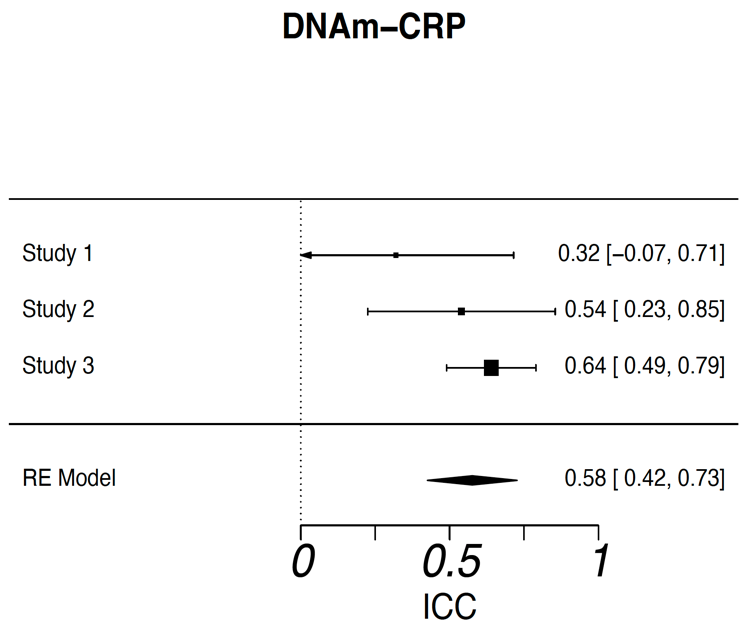 |
| **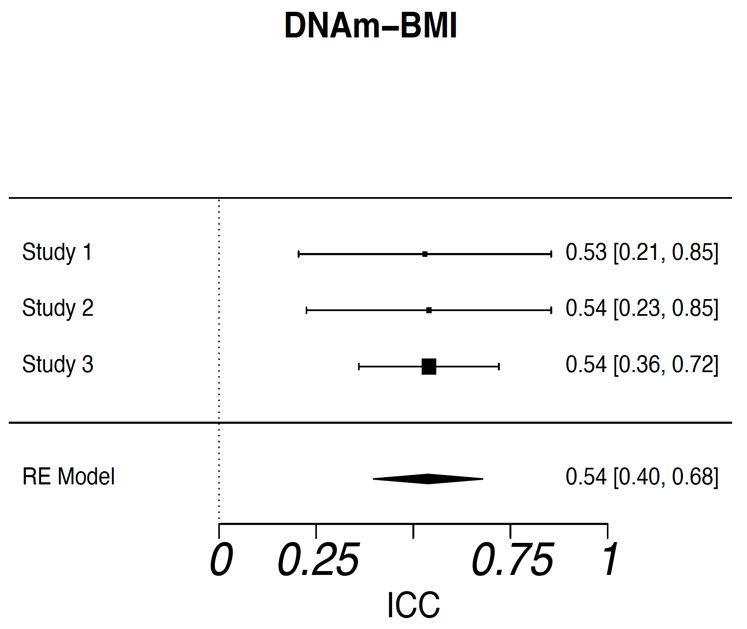** | **** |
| **Supplementary Figure 2. Forest plot of within-dataset (study) meta-analysis ICCs for each DNA methylation profile score corrected for cell composition using the reference free cell estimation method:** Study 1 (n=21), Study 2 (n=22) and Study 3 (n=64). "Skin Horvath" denotes the Horvath Skin and Blood epigenetic clock. | |
